# Catheter Event Rates in Medical Compared to Surgical Peritoneal Dialysis Catheter Insertion

**DOI:** 10.1016/j.ekir.2023.09.015

**Published:** 2023-09-17

**Authors:** James Fotheringham, Ivonne Solis-Trapala, Victoria Briggs, Mark Lambie, Keith McCullough, Louese Dunn, Andrew Rawdin, Harry Hill, Allan Wailloo, Simon Davies, Martin Wilkie

**Affiliations:** 1 Sheffield Kidney Institute, Sheffield Teaching Hospitals NHS Trust, Sheffield, UK; 2 School of Health and Related Research, University of Sheffield, Sheffield, UK; 3 School of Medicine, Faculty of Medicine and Health Sciences, Keele University, Staffordshire, UK; 4 Arbor Research Collaborative for Health, Ann Arbor, Michigan, USA

**Keywords:** catheter insertion, dialysis access, peritoneal dialysis

## Abstract

**Introduction:**

How patient, center, and insertion technique factors interact needs to be understood when designing peritoneal dialysis (PD) catheter insertion pathways.

**Methods:**

We undertook a prospective cohort study in 44 UK centers enrolling participants planned for first catheter insertion. Sequences of regressions were used to describe the associations linking patient and dialysis unit-level characteristics with catheter insertion technique and their impact on the occurrence of catheter-related events in the first year (catheter-related infection, hospitalization, and removal). Factors associated with catheter events were incorporated into a multistate model comparing the rates of catheter events between medical and surgical insertion alongside treatment modality transitions and mortality.

**Results:**

Of 784 first catheter insertions, 466 (59%) had a catheter event in the first year and 61.2% of transitions onto hemodialysis (HD) were immediately preceded by a catheter event. Catheter malfunction was less but infection was more common with surgical compared with medical insertions. Participants at centers with fewer late presenters and more new dialysis patients starting PD, had a lower probability of a catheter event. Adjusting for these factors, the hazard ratio for a catheter event following insertion (medical vs. surgical) was 0.70 (95% confidence interval [CI] 0.43 to 1.13), and once established on PD 0.77 (0.62 to 0.96).

**Conclusion:**

Offering both medical and surgical techniques is associated with lower catheter event rates and keeps people on PD for longer.

## Graphical abstract

**Figure:**
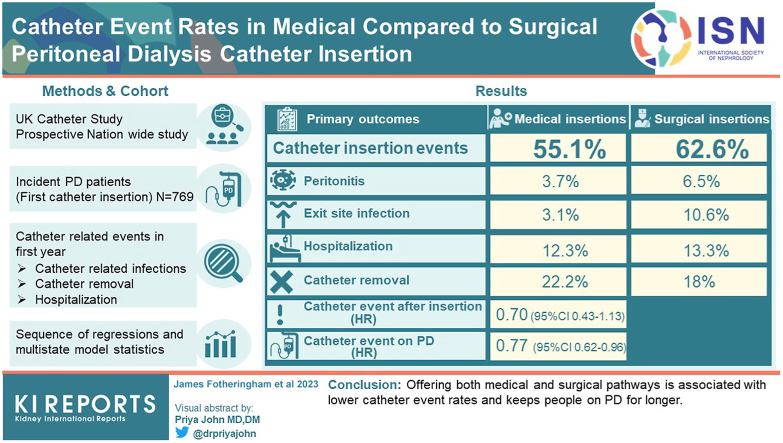

High quality peritoneal access is essential for effective PD; and in clinical practice, it is influenced by multiple factors such as patient and center characteristics, local availability of resources and expertise, as well as organizational aspects from a health service perspective.^1^ Inclusive care pathways require approaches that enable the insertion of timely and effective PD access for individuals who are unsuitable for general anesthesia, or who have had previous significant abdominal surgery. The recent COVID-19 pandemic has emphasized the need for responsive access pathways, including those that can accommodate starting urgently on PD, which are known to have a beneficial effect on the use and uptake of PD as a therapy.^2^ This responsiveness should not be at the expense of more access complications that have a deleterious effect on early technique survival.^3^

Several operative methods are described for PD catheter insertion, and these can be broadly classified into medical (percutaneous, radiological, and peritoneoscopic) or surgical (open surgical and laparoscopic). Although there is little evidence to support the use of one technique over another,^4^ a systematic review of cohort studies in 2018 suggests that clinical outcomes are better when advanced techniques (including rectus sheath tunnelling and adjunctive procedures) are combined with laparoscopic insertion when compared with basic laparoscopy or open surgical insertion.^5^ Equally, percutaneous insertion may be associated with lower complication rates of tunnel and exit site infections^6^ although this is not certain. International registries collecting data on catheter insertion techniques and practice patterns have shown wide variations in event rates and catheter survival.^7^, ^8^, ^9^ However catheter insertion is as much about the care pathway as the insertion technique, and it is not clear whether there are clinical advantages or disadvantages to a PD program in offering both medical and surgical pathways, other than that of increased access to the therapy.^2^^,^^10^

We aimed to examine how patient-level and center-level factors influence the choice of catheter insertion technique for a PD patient, and the impact of such factors and pathways on the occurrence of catheter events, with a primary aim of comparing the rates of catheter-related events between medical and surgical insertion techniques considering the patient’s treatment history and mortality. To do this, we undertook a UK-wide, multicenter prospective cohort study to establish how patient clinical history, and dialysis unit-level characteristics and practices impacted on the subsequent occurrence of adverse catheter-related events within 12 months of follow-up. We utilized sequences of regressions, a subclass of graphical models, to describe concisely through a visual representation, the associations that link center and patient related characteristics with catheter insertion technique and their impact on the probability of catheter-related events. From this, we chose relevant factors to include in a multistate model of the patient journey from catheter insertion through catheter events and modality changes to transplantation and death, with the goal of estimating the impact of the catheter insertion technique on the rate of catheter-related events, accounting for relevant factors. We anticipate that our findings will inform dialysis units’ decisions to either focus their efforts on improving a single surgical pathway or to utilize a combination of surgical and medical insertion pathways.

## Methods

### Design and Study Population

UK Cath was a prospective multicenter cohort study of incident PD patients at first catheter insertion conducted in 44 of the 72 dialysis centers in the UK, 20 of which were simultaneously participating in the Peritoneal Dialysis Outcomes and Practice Patterns Study (PDOPPS).^11^ The protocol was published in 2017, prior to study closure and analysis.^12^ People with irreversible kidney failure ≥18 years of age intending to begin PD as their first modality and who planned to undergo PD catheter insertion within the next 30 days were offered the opportunity to consent for the study. Patients were excluded if they had already begun PD, had their PD catheter inserted at a different center, or if they were unable to give informed consent. Recruitment commenced in July 2015 and finished at the end of December 2017.

The study was sponsored by Sheffield Teaching Hospitals NHS Foundation Trust and Ethical committee approval for the study was granted by the National Research Ethics Committee London – City and East in December 2013 (Ref: 13/LO/1943). All subjects received a patient information sheet and gave written consent. Patient partners were involved at each stage of the research process, including the design of patient-facing questionnaires.

### Instrument Development, Data Collection, and Data Sources

Survey instruments from PDOPPS were adapted for use in the United Kingdom and for this specific study. The development, trialing, data collection methods, and supplemental data sources are described in the Supplementary Material.

### Primary Outcome

The primary outcome measure was the occurrence of a catheter-related event, captured using a catheter event worksheet or catheter-related hospitalization codes or infection events from the 4-monthly interval summary. PD catheter-related events were defined in the protocol as 1 or more of the following: further operative procedures or catheter removal (as a consequence of dialysis fluid leak, hernia, poor or absence of flow, catheter displacement, bleeding, catheter-related pain, exit site infection, tunnel infection, wound infection, or peritonitis [See Supplementary Item, Catheter Event Worksheet]), catheter-related infections not requiring a procedure, or hospital admission for catheter-related events as listed above (Supplementary Table S2) as well as mechanical failure of function leading to treatment disruption >3 days duration or the need for HD.

### Postulated Patient-Level and Center-Level Factors Contributing to Catheter-Related Events

**Figure 1:**
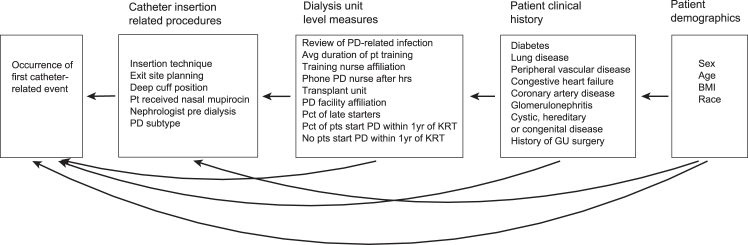
Graphical representation of postulated *a priori* model of pathways of associations in which 4 domains, including patient demographics, patient clinical history, dialysis unit-level measures, and catheter insertion related procedures lead to the occurrence of a catheter-related adverse event within 1 year. The variables on the left are regarded as responses to those located to their right and any 2 variables in different domains are linked by arrow lines or sequences of connected arrow lines indicating potential directed or indirect associations respectively. The association between 2 variables located in different boxes can be directed (if linked by an arrow line) or explained by intermediate factors that are located in boxes between them in the graph (if linked by a sequence of connected arrow lines). BMI, body mass index; GU, genitourinary; KRT, kidney replacement therapy; PD, peritoneal dialysis; Pt, patient.

Our analysis was informed by the postulated direction of associations depicted in Figure 1, where the occurrence of a first catheter-related event appears within a box on the left-hand side and is a response variable to the 4 domains also grouped in boxes and placed on the right; arrow lines are used to indicate that variables within each box depend in principle on all variables to their right. The order of the domains in the graph was chosen first, to assess whether the probability of a catheter event was associated with catheter insertion related procedures when controlling for patient-level and center-level factors. Second, to identify the primary factors leading to catheter events directly or indirectly via intermediate factors. The variables included in the postulated model are described in Supplementary Table S1.

### Statistical Analysis

The statistical analysis used sequences of regressions, a subclass of graphical models, to describe the direct associations of patient demographics and clinical history, and indirect associations via center-level factors with catheter insertion related procedures and their impact on the probability of the first catheter-related event within 12 months follow-up.^13^^,^^14^ The factors that were associated with the probability of a catheter-event were then incorporated into a multistate model to compare the rates of catheter-related events, alongside treatment modality transitions and mortality.

#### Sequences of Regressions

The model was built by fitting ordered sequences of logistic regression models for binary outcomes and linear regression models for continuous outcomes, for each variable in the different groups of variables. Each variable in turn, starting with the occurrence of a catheter-related event and moving from left to right (Figure 1), was fitted into a regression model with all the variables to its right-hand side as explanatory. Patient-level variables were clustered within a center; a random intercept was added to their regression model to account for the correlation between observations from patients treated in the same center. We also estimated the pairwise association between the “number of patients starting PD within a year of kidney replacement therapy (KRT) initiation”, “percentage of patients starting PD within 1 year of KRT initiation” and “percentage of late starters” because these are closely related measures that were associated with the occurrence of a catheter event.

**Figure 2:**
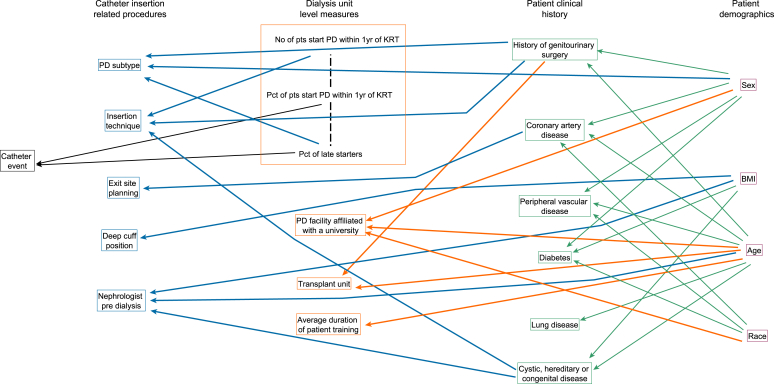
Regression graph for sequences of regressions model of best fit. This graph disentangles the relative importance of patient-level and center-level factors on catheter insertion procedures, and the occurrence of a catheter-related-event within 1 year. An arrow line emerging from an explanatory variable and pointing to a response variable of the same color represents a direct association, controlling for all its remaining regressors. A sequence of connected arrow lines between 2 variables represents an indirect association. Direct associations are highlighted using black, blue, orange, and green arrow lines for factors associated with catheter events, catheter insertion related procedures, dialysis unit level measures, and patient clinical history, respectively. A dashed line was used to indicate a significant undirected association quantified by a Pearson correlation coefficient between a pair of variables characterizing the number of patients starting PD within 1 year of KRT, percentage of patients starting PD within 1 year of KRT and percentage of late presenters in center. These variables are placed within a box in the graph, whereas all other variables are presented in stacked boxes within their group categories because undirected associations were not estimated for the latter. KRT, kidney replacement therapy; Pct, percent; PD, peritoneal dialysis.

The sequences of regressions are described using a regression graph (Figure 2) in which 2 variables located in different groups were linked by an arrow line emerging from a selected explanatory variable and pointing to a response variable if they were directly associated. The strength of this association was quantified with a partial regression coefficient. In the fitted model, all the response variables which had at least 1 important explanatory variable were binary; therefore, odds ratios (ORs) and 95% CIs were reported. A sequence of connected arrow lines between 2 variables represents an indirect association. These variables are placed within a box in the regression graph whereas all other variables are presented in stacked boxes within their group categories to make this distinction in Figure 2.

#### Multistate Model

**Figure 3:**
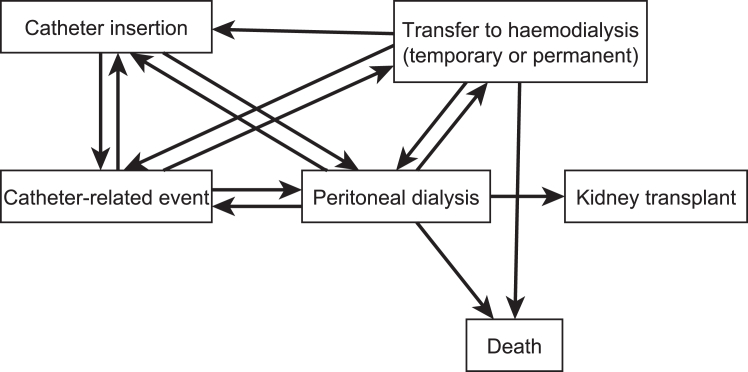
Multistate model for time-to-event outcomes. The model allows 14 transitions represented by 14 arrows connecting states in the figure. For example, a patient may have a catheter-related event following either a catheter insertion, PD or HD as indicated by the 3 arrow lines emerging from the latter states and pointing to catheter insertion. Patients who had a kidney transplant or died were no longer observed in the study; therefore, these states have no outgoing arrows. HD, hemodialysis; PD, peritoneal dialysis.

The multistate model (Figure 3) analyzed the patient event history, characterized by the following 6 states: catheter insertion, catheter-related event, PD, temporary or permanent transfer to HD, kidney transplant, and death. Each patient had a sequence of transitions from one state to another coupled with the time (days from baseline) of transition. In Figure 3, we show the 6 states and 14 transitions allowed by the model. Patients who had a kidney transplant ceased follow-up in the UK Catheter study and therefore this state and death have no outgoing arrow lines. The primary parameters of interest were the rates of transitions (i.e., the hazard of moving from one state to another) from catheter insertion to catheter-related event and PD to catheter-related event. A time-varying binary variable “catheter insertion technique” was created to indicate what insertion technique was used (medical or surgical) for a catheter that had an event and included *a priori*.

The hazard functions of transitions from the states of catheter insertion and PD to catheter event were modelled in terms of catheter insertion technique and factors that were found to be directly associated with the probability of a catheter-related event within a year in the sequences of regressions model. Hazard ratios and 95% CIs were reported for each explanatory variable. The hazard functions for the transitions between the PD state and the states of kidney transplant and death included age, race, sex, and comorbidities as explanatory variables, with body mass index added to the hazard functions for the transitions to death. The model fitted each time to transition separately by maximum likelihood estimation, assuming a Weibull distribution and the semi-Markov property, whereby the probability of moving from one state to another depends on the time since entry into the current state.

Catheters in which insertion technique was unknown were excluded from the analysis. Baseline characteristics were described by catheter insertion technique using frequencies (%) and means (SD) for categorical and continuous variables, respectively.

To explore the relative importance of the informing events of the composite primary outcome, in addition to reporting the frequency of these first events in the first 12 months of follow-up, the proportions of these informing events in patients who had a catheter event in the 2 weeks before transitioning onto in-center HD was compared. This was deemed the most negative transition for a person receiving a home dialysis therapy, and it was hypothesized that differences in these proportions might reveal a more significant component of the primary end point, which is masked by earlier more frequent but less deleterious events.

Missing data across the variables range from 1.4% to 35.3% (median 5.1%, interquartile range 2.3% to 9.3%) used in the sequences of regression analyses. To address this issue, missing patterns were examined; expectation-maximization imputation was used, which preserves the covariance of the data to obtain estimates that make effective use of all the data; and sensitivity analyses were carried out. Further details on model estimation, assumptions, dealing with missing data, goodness of fit, and diagnostic checks for the sequences of regressions and multistate models are provided in the Supplementary Statistical Methods. The level of statistical significance was set at 0.05, and all analyses were performed using the packages “lme4,” “flexsurv,” and “mitml” in the statistical software R (R Foundation for Statistical Computing, Vienna, Austria).^15^, ^16^, ^17^, ^18^

## Results

### Participating Patient and Center Characteristics, and Catheter Insertion Technique

After obtaining consent from 837 patients, 784 first catheter insertions were recorded. Information about catheter insertion technique was missing for 15 participants, leaving 769 for the descriptive analysis whose baseline characteristics are presented in Table 1, totaling 564 patient-years of follow-up. Patient and center recruitment and retention in the analysis are detailed in Supplementary Figure S1. Of the patients, 325 (42%) received medical insertions and 444 (58%) received surgical insertions. Of White patients, 91% had surgical insertions compared to 80% of those of non-White ethnicity. Of those with a history of abdominal, genitourinary, or gastrointestinal surgery, 29% had a surgical insertion compared to 16% of those who had not. The prevalence of congestive heart failure was 8.1% in medical catheter insertions and 4.7% of surgical insertions; however, other comorbidities including diabetes were equally distributed between medical and surgical insertion techniques. Comparison of insertion techniques between this study and contemporaneous data from the UK Renal Registry is presented in Supplementary Figure S2.

**Table 1:** Patient baseline and dialysis unit practices by catheter insertion technique

Characteristics	Medical insertion (*n* = 325)	Surgical insertion (*n* = 444)	Total (*N* = 769)^a^	Missing (%)^b^ 15 (1.9)
Patient demographics				
Sex, female (%)	100 (31.7)	164 (37.2)	264 (34.9)	14 (1.8)
Age, mean yrs (SD)	59.0 (16.7)	58.3 (15.6)	58.6 (16.0)	14 (1.8)
Body mass index, mean kg/m^2^ (SD)	27.3 (5.4)	27.8 (5.0)	27.6 (5.2)	71 (9.1)
Race, *n* (%)				16 (2.0)
White	252 (80.0)	399 (90.9)	651 (86.3)	
Other	63 (20.0)	40 (9.1)	103 (13.7)	
Patient clinical history				
Comorbid conditions, *n* (%)				74 (9.4)
Diabetes	94 (31.9)	127 (31.4)	221 (31.6)	
Lung disease	15 (5.1)	20 (5.0)	35 (5.0)	
Peripheral vascular disease	20 (6.8)	36 (8.9)	56 (8.0)	
Congestive heart failure	24 (8.1)	19 (4.7)	43 (6.2)	
Coronary artery disease	49 (16.6)	61 (15.1)	110 (15.7)	
Primary cause of end-stage renal disease, *n* (%)				154 (19.6)
Glomerulonephritis	42 (16.4)	74 (20.4)	116 (18.7)	
Cystic, hereditary, or congenital disease	10 (3.9)	51 (14.0)	61 (9.9)	
Abdominal, genitourinary, or gastrointestinal surgery (%)	48 (16.3)	116 (28.7)	164 (23.5)	74 (9.4)
Catheter insertion related procedures				
Exit site planning was formally documented prior to catheter insertion? yes (%)	216 (74.7)	293 (74.0)	509 (74.3)	87 (11.1)
Deep cuff position, *n* (%)				48 (6.1)
central or midline	272 (86.1)	314 (76.4)	586 (80.6)	
paramedian	44 (13.9)	97 (23.6)	141 (19.4)	
Nasal mupirocin prior to the procedure (%)	121 (37.9)	138 (32.6)	259 (34.9)	34 (4.3)
How long patient saw nephrologist before start of chronic dialysis, more than 6 months (%)	138 (75.4)	234 (74.1)	372 (74.5)	277 (35.3)
PD subtype, *n* (%)				136 (17.4)
APD	114 (44.5)	186 (48.8)	300 (47.1)	
CAPD	142 (55.5)	195 (51.2)	337 (52.9)	
Dialysis unit level measures				
Review rates of PD-related infections for quality improvement, *n* (%)				46 (5.9)
Every 1–3 mo	138 (43.1)	115 (28.4)	253 (34.9)	
Every 3–6 mo	37 (11.6)	52 (12.8)	89 (12.3)	
Every 6–12 mo	98 (30.6)	114 (28.1)	212 (29.2)	
Every 1–2 yrs	33 (10.3)	46 (11.4)	79 (10.9)	
Never	14 (4.4)	78 (19.3)	92 (12.7)	
Average duration of patient training prior to PD initiation, *n* (%)				52 (6.6)
2–3 d	124 (41.3)	202 (48.3)	326 (45.4)	
4 or more d	176 (58.7)	216 (51.7)	392 (54.6)	
Affiliation of nurses who initially train patients				11 (1.4)
All employed at facility	207 (64.5)	281 (64.2)	488 (64.3)	
Combined: facility and third party	114 (35.5)	157 (35.8)	271 (35.7)	
PD nurses contactable by phone outside working hours (%)	144 (44.9)	224 (51.1)	368 (48.5)	11 (1.4)
Transplant unit, yes (%)	73 (23.5)	151 (34.9)	224 (30.1)	26 (3.3)
PD facility affiliated with a university, yes (%)	197 (64.2)	170 (39.2)	367 (49.5)	29 (3.7)
Percentage of late starters in center, mean (SD)	16.0 (4.3)	16.3 (3.5)	16.2 (3.9)	56 (7.1)
Percentage of patients starting PD within 1 year in center, mean (SD)	25.2 (8.9)	23.9 (8.2)	24.4 (8.5)	26 (3.3)
No of patients starting dialysis per year in center, mean (SD)	130.6 (55.0)	88.4 (46.5)	106.0 (54.3)	26 (3.3)

APD, automated PD; CAPD, continuous ambulatory PD; PD, peritoneal dialysis.

a Denominators vary because the variables have different completeness rates.

b Number (%) of missing values for each variable, the median percentage of missing data was 3% (interquartile range: 6.4%–9.4%).

Of patients who had medical catheter insertion, 43% were treated in centers that reviewed their infection rates more frequently than every 3 months, compared to 28% of patients receiving surgical insertions. Of patients receiving a medical insertion, 4% were treated in centers that never reviewed infection rates, compared to 19% of patients receiving a surgical insertion. Participants who had medical insertions were treated in centers that started a mean of 130 patients on PD per year and 64% were associated with a university, compared to a center mean of 88 patients on PD per year and 39% of centers being associated with a university for those patients receiving surgical insertions.

### The Associations of Catheter Insertion Technique, Patient-Level, and Center-Level Characteristics With Catheter Events

#### Patient and Center Factors Influencing Catheter-Related Insertion Technique

The regressions graph for catheter events corresponding to the model that best fitted the data is shown in Figure 2. For greater clarity, the graph was divided into 3 subgraphs (Supplementary Figures S3–S5) displaying the strength of associations. The main findings are summarized working through each group of variables, from left to right below. More narrative detail on dialysis unit level measures and patient history is presented in Supplemental Materials.

The sequences of regressions estimated the odds of a medical catheter insertion were lower in individuals with a primary diagnosis of cystic, hereditary, or congenital disease (OR 0.12, 95% CI 0.04 to 0.36), or a history of abdominal, genitourinary, or gastrointestinal surgery (OR 0.28, 95% CI 0.14 to 0.56, with additional interpretation in Supplementary Materials). Age and sex were indirectly associated with the choice of insertion technique through their association with patient’s history of genitourinary surgery, and medical insertion was positively associated with the number of patients starting PD within 1 year of KRT initiation.

#### Patient, Center, and Insertion Technique Factors Influencing Catheter-Related Events Within 1 Year

A total of 466 (60.6%) of catheter insertions had an event during the first year of follow-up, of which 179 of 325 (55.1%) followed medical insertions and 278 of 444 (62.6%) surgical insertions, as shown in Table 2, which includes the cause of the event. For all catheter insertions, 29.5% had catheter function events, 5.5% had peritonitis, 7.0% had an exit site infection, and 12.5% had a catheter-related hospitalization. Catheter failure, defined as a catheter removal within a year, not performed as a result of noncatheter-related modality transition or recovery of renal function, was 72 of 325 (22.1%) in the medical arm and 80 of 444 (18.0%) in the surgical arm.

**Table 2:** Frequency (%) of first catheter-related event within 1 year from insertion

Event descriptor	Medical	Surgical	Total
Insertions with an event	179 (55.1)	278 (62.6)	457 (59.4)
**Cause of event:**			
Catheter function	110 (33.8)	111 (25.0)	221 (28.7)
Peritonitis	12 (3.7)	29 (6.5)	41 (5.3)
Exit site infection	10 (3.1)	47 (10.6)	57 (7.4)
Hospitalization	40 (12.3)	59 (13.3)	99 (12.9)
More than one cause	7 (2.2)	32 (7.2)	39 (5.1)
Catheter Failure (removal)	72 (22.2)	80 (18.0)	152 (19.8)
Insertions with no event	146 (44.9)	166 (37.4)	312 (40.6)
Total number of insertions	325 (100)	444 (100)	769 (100)

The sequences of regressions which estimated the occurrence of a catheter event was directly explained by 2 center-level characteristics, the odds of a catheter event were lower if the percentage of patients receiving PD within 1 year of KRT initiation was greater (OR 0.97, 95% CI 0.94 to 1.00) and higher if the percentage of patients presenting late was greater (OR 1.07, 95% CI 1.00 to 1.14) (Supplementary Table S3 and Supplementary Figure S3, with additional interpretation in Supplementary Materials). There were no other insertion, patient-level or center-level characteristics influencing catheter events that were not explained by these 2 factors. The estimates of the relationship between patient characteristics, clinical history and centre level factors are presented in Supplementary Tables S4 and S5. The sequences of regressions did not identify a statistically significant association between medical compared to surgical catheter insertion and catheter event; however, this method was designed and powered to describe the context and inform the multistate model rather than test this hypothesis.

### Multistate Model

Catheter insertion was the starting state for 96% of the cohort. In Supplementary Table S6, we show the observed frequency of movements from one state to another at successive observation times. There were 201 movements from catheter insertion to catheter event in 189 patients, with 6% of participants having 2 to 3 transitions. There were 673 transitions from PD to catheter events in 381 patients with 24% of participants having 2 transitions and 19% having 3 to 7 multiple transitions. The catheter event rate from insertion was constant over time, whereas from PD it was greater in the first 3 months (Supplementary Figure S6, which also illustrates model fit). Of transitions to HD, 61.5% (156/255) were preceded within 2 weeks by a catheter event. The type of catheter event which caused this transition overall and stratified by insertion technique is reported in Table 3.

**Table 3:** Frequency (%) of causes of catheter event in patients transitioning from catheter event to in-center hemodialysis

Cause of event before transition onto hemodialysis	Medical	Surgical	Total
Catheter Function	20 (42.6%)	53 (52.0%)	73 (49.0%)
Peritonitis	4 (8.5%)	15 (14.7%)	19 (12.8%)
Exit Site Infection	2 (4.3%)	10 (9.8%)	12 (8.1%)
Hospitalization	17 (36.2%)	21 (20.6%)	38 (25.5%)
More than 1 cause	4 (8.5%)	3 (2.9%)	7 (4.7%)
Total	47 (100%)	102 (100%)	149 (100%)

In Table 4, we show the estimated hazard ratios for transitions into catheter event, and from PD to transplantation and death. The hazard ratio for medical catheter insertion versus surgical was 0.70 (0.43 to 1.13) for the transition from catheter insertion to catheter event and 0.77 (0.62 to 0.96) for the transition from PD to catheter event, adjusting for percentages of patients starting PD within 1 year of KRT initiation and late presenters.

**Table 4:** Estimated hazard ratios for selected multistate model transitions

Covariate	Catheter insertion to catheter-related event, model (b) HR (95% CI)	PD to catheter-related event, model (b) HR (95% CI)
Catheter insertion, medical	0.70 (0.43, 1.13)^a^	0.77 (0.62, 0.96)^b^
Dialysis unit measures		
Percentage of late presenters	1.05 (0.99, 1.11)	1.02 (0.99, 1.05)
Percentage of patients starting PD within 1 year of KRT initiation	0.96 (0.93, 0.99)	0.99 (0.97, 1.00)

	PD to kidney transplant HR (95% CI)	PD to death HR (95% CI)
Age in years	0.97 (0.96, 0.98)	1.04 (1.02, 1.06)
Comorbid conditions		
Lung disease, yes		2.69 (1.11, 6.49)
Peripheral vascular disease, yes		2.21 (1.14, 4.25)
Congestive heart failure, yes	0.26 (0.08, 0.83)	
Coronary artery disease, yes		2.33 (1.40, 3.90)

CI, confidence interval; HR, hazard ratio; KRT, kidney replacement therapy; PD, peritoneal dialysis.

Models for transitions from catheter insertion to catheter-related event and PD to catheter-related event, compared the transition rates between medical and surgical insertions.

a Estimated hazard ratio from model including catheter insertion as explanatory variable only: 0.76 (0.47, 1.22).

b Estimated hazard ratio from model including catheter insertion as explanatory variable only: 0.77 (0.63, 0.94).

## Discussion

In this study we report the most comprehensive analysis of the PD catheter insertion pathway to date. Our sequence of regressions model comprehensively describing the insertion pathway showed in this representative UK national cohort that the most important and significant factors increasing the risk of an adverse catheter-related outcome were a lower percentage of patients receiving PD and a higher number of late-presenters within a dialysis center. Our multistate modeling demonstrated superior clinical outcomes with medical insertion technique, because the transition from PD to a catheter event was associated with reduced risk compared to surgical insertion.

We found that approximately half of the participating centers offered a medical pathway, in keeping with national registry data.^19^ Centers using both insertion pathways were larger, undertook more infection-related audit, were more likely to be affiliated with a university (but less likely to be a transplanting unit). This finding fits well with a growing body of evidence that the volume of PD-related activity within a dialysis center equates to better clinical outcomes. Several studies, including analyses of data from registries and dialysis networks, as well as a systematic review, indicate that low center PD program size is a risk for transfer to HD (formerly referred to as technique failure).^20^, ^21^, ^22^, ^23^ The ANZDATA group found that the percentage of patients using PD and center size (which may only be a surrogate for the former based on our analysis) were associated with reduced risk of transfer to HD.^24^ This strongly suggests that it is center-level experience and organization rather than patient mix that is important, because centers that offer PD to a larger percentage are likely to be more inclusive of those at higher risk. Late presentation to a dialysis unit is a known risk factor for worse survival and reduced access to PD, and we now show that some of this is mediated in part by worse catheter outcomes.^25^^,^^26^ A recent multicenter study of complications following laparoscopic insertion documented by the North American PD Catheter Registry found that 24% were associated with adverse events by 6 months,^8^ comparable to the 12-month combined risk of mechanical and hospitalized catheter events of 38.3% in our surgical pathway analysis. Despite the inclusion of these events, we found that the number of catheter removals with each insertion technique was similar. It may be relevant that 65% of catheters in that study had a deep cuff located in the rectus sheath compared with 19.4% being located in a paramedian position in our study.

Our study has the strength that it describes and considers the influence of aspects of the clinical pathway which increasingly has been recognized as important,^8^^,^^27^ whereas previous studies have tended to focus on insertion technique rather than the pathway.^5^^,^^28^ To our knowledge, this is the first study to employ graphical and multistate modeling, capable of describing the complex interrelationship of the different steps of achieving catheter function and remaining on PD, to evaluate both clinical and cost-effectiveness (which will be presented in a separate manuscript). The UK Catheter study has a number of limitations. It is an observational cohort study of real practice rather than what would ideally be a cluster randomized controlled trial. There may be residual confounding and our attempts to infer cause and effect must be treated with caution. The use of sequences of regressions ordered according to expected cause and effect relationships attempts to minimize this problem but cannot be considered perfect. We chose to evaluate a composite outcome, as opposed to a single measure of catheter function (e.g., catheter removal), driven by a desire to describe this problem holistically and include within the outcome other events shown to be important to PD patients (i.e., infection and transfer to HD).^29^ We did not have sufficient numbers to address subtypes of catheter insertion technique (e.g. laparoscopic insertion), examine their effect on pathways separately, or confidently conclude that medical compared to surgical insertion was superior from catheter insertion to catheter event. There was a moderate amount of missing data across the variables used in the sequences of regression analyses; however, extensive sensitivity analyses showed unbiased parameter estimates. Importantly, this study was conducted within a single healthcare system and should be generalized to other systems with caution.

This study has implications for healthcare policy and practice. It is well recognized that the decision by clinical teams to set up an alternative “medical” insertion pathway in addition to an existing surgical catheter insertion pathway is largely driven by the advantages this has to offer in terms of local organizational constraints (e.g., lack of access to operating rooms, surgeon availability, and waiting times) and the need to start patients on dialysis urgently, rather than a perceived benefit in terms of clinical outcomes. It is ubiquitous for medical catheter insertions to be supported by a surgical catheter insertion service. Our study provides reassurance about the medical insertion approach, important because earlier UK Renal Registry data had suggested that medical insertion may be less successful.^30^ There is a suggestion from our data that surgical insertion is less likely to lead to mechanical problems and vice versa for infection, which may reflect the relative strengths of each insertion technique at different stages of the associated clinical pathway. The percentage of people of non-White ethnicity receiving PD in centers using surgical insertion only was only 4.3% compared to 20% in those offering both pathways and this should be further investigated as a potential barrier to PD access.^31^

In summary, the UK Catheter study has significant implications for the design of PD catheter insertion pathways. It builds on previous evidence that having a typically physician-led “medical” pathway increases access to PD by showing that outcomes are similar or better using this approach when taking a holistic perspective. It seems likely that these better outcomes reflect the fact that medical catheter insertion techniques are used by centers that have a stronger PD focus and greater experience of the modality.

## Disclosure

JF and MW have received speaker honoraria from Fresenius Medical Care and have conducted research funded by Baxter. ML has received speaker honoraria from Baxter Healthcare and Fresenius Medical Care; and a research grant from Baxter Healthcare in 2013. SD has received speaker honoraria from Fresenius Medical Care, research funding from Baxter Healthcare, and advisory board fees from Baxter HealthCare and Ellen Medical. All other authors have declared no conflicting interest.

## Supplementary Material

Supplementary File (PDF)

**Text 1.** Supplemental study methods.

**Text 2.** Supplemental sequences of regressions modeling.

**Text 3.** Supplemental multistate model methods.

**Text 4.** Supplemental interpretation of results from the sequences of regressions modeling.

**Figure S1.** CONSORT diagram

**Figure S2.** External generalizability PD catheter insertion techniques

**Figure S3.** Regression subgraph for catheter-related event as response variable.

**Figure S4.** Regression subgraph for catheter insertion related procedures.

**Figure S5.** Regression subgraph for dialysis unit measures and patient clinical history.

**Figure S6.** Cumulative hazard functions of selected multistate transitions.

**Table S1.** Description of variables used in the initial sequences of regressions model (Figure 1)

**Table S2.** Primary hospitalization codes for catheter events purely relating to hospitalization.

**Table S3.** Models describing direct associations for catheter-related event and catheter insertion procedures as response variables.

**Table S4.** Models describing direct associations for dialysis unit level measures as response variables.

**Table S5.** Models describing direct associations for clinical history as response variables.

**Table S6.** Table of observed transitions described in Figure 3.

**STROBE Statement**.
