# Supplementary material for: Catheter Event Rates in Medical Compared to Surgical Peritoneal Dialysis Catheter Insertion
Source: Kidney Int Rep. 2023 Sep 17;8(12):2635–45. doi: 10.1016/j.ekir.2023.09.015 (PMC10719604; doi:10.1016/j.ekir.2023.09.015)
Supplement: Supplementary File (PDF) [file mmc1.pdf]

Catheter event rates in medical compared to surgical peritoneal dialysis catheter insertion - Supplemental Materials

Contents

Procotol paper 2

Supplementary Text 1: Supplemental study methods 2

Study governance 2

Process of Patient Informed Consent 2

Investigator meetings 2

Constitution of the study advisory group. 2

External generalization and the basis of the STROBE diagram (Supplemental Figure 1) 3

Supplemental methods 4

Instrument development, data collection and data sources 4

Supplementary Text 2: Supplemental sequences of regressions modelling 5

Supplementary Text 3: Supplemental multistate model methods 7

Supplementary Text 4: Supplemental interpretation of results from the sequences of regressions modelling 9

Factors influencing catheter-related events within 1 year 9

Catheter insertion related procedures 9

Dialysis unit level measures 9

Patient clinical history 10

Supplemental figures 10

.... 10

Supplemental Tables 16

UK Cath Data Collection Worksheets 22

Catheter Event Worksheet 22

Catheter Insertion Worksheet 25

Catheter care and follow-up form 30

STROBE Statement—Checklist of items that should be included in reports of *cohort studies* 33

Procotol paper

Perit Dial Int 2018; 38(2):113–118 epub ahead of print: 28 Sept 2017 (1)

https://doi.org/10.3747/pdi.2017.00083

Supplementary Text 1: Supplemental study methods

Study governance

Study governance and ethics submissions were supervised by LD from Sheffield including the submission of ethics committee amendments. The study was adopted onto the National Institute for Health Research (NIHR) portfolio (UKCRN ID 17940) which enabled support from NIHR research nursing and was supported by a dedicated study website that held study instruments and the protocol as well as study management information. The study co-ordinator supervised centre participation on a daily basis, visiting centres during the set-up phase, provided training in the use of PDOPPSLink responded to data queries, managing the consent recruitment log and consent forms. Patients were followed until the end of the study, permanent transfer to HD (defined as 4 months on HD), transplantation or death. This analysis includes 1 year follow-up data - completeness ranged according to domain e.g. 100% for operator to 63% whether antibiotics were given pre-operatively.

Process of Patient Informed Consent

An appropriately delegated staff member sought informed consent from incident and prevalent patients. The consent process began at the time of assessment for PD catheter insertion with a plan to obtain study consent before the time of catheter insertion subject to meeting eligibility requirements. For recruited patients who were at home at the time of selection, tentative agreement was initially obtained via communication from the study coordinator (i.e., by phone or email). Consent was then confirmed via completion of the signed consent form.

Investigator meetings

Investigator meetings were held in Sheffield in September 2015 and December 2016 and steering groups were conducted 2 – 3 times per year locates either in Sheffield or Stoke.

Constitution of the study advisory group.

In addition to co-authors the advisory group included

- Mr Badri Shrestha consultant transplant and access surgeon Sheffield
- Dr Sarah Jenkins, consultant nephrologist Sheffield.
- Alina Andras – study co-ordinator
- Yvonne Jackson – research nurse Sheffield Teaching Hospitals.
- Dr Lin Wang – patient partner
- Dr Louise Phillips-Darby - Clinical Studies Manager, Keele University.
- Stephanie MacNeill UK Renal Registry
- Prof Fergus Caskey, UK Renal Registry
- Prof Helen Hurst, Consultant Nurse, Manchester University Hospitals NHS Foundation Trust
- Cheryl Bailey, study co-ordinator, Sheffield Teaching Hospitals.
- Dr Jean Winterbottom, Research Nurse, Sheffield Teaching Hospitals.
- Dr Nana Theodorou, Research Co-ordinator, Sheffield Teaching Hospitals.

- Dr John Belcher, Senior Statistician, Keele University.
- Jodi Paget, Research Nurse, Bradford Hospitals
- Diane Palframa, research nurse, Bradford Hospitals.
- Karen Chambers, Jennifer King *DOPPS coordinators for the UK*

External generalization and the basis of the STROBE diagram (Supplemental Figure 1)

	UK Renal Registry Data			UK Cath study
	**2015**	**2016**	**2017**	**2015-2017 47 centres with total of 737 months**
Incident Dialysis	7,869	7,797	8,001	
% PD	19.30%	20.30%	19.30%	
Incident PD	1519	1583	1544	1,370
Mean Age			63.7	58.6
% Male			64.10%	65.10%
% White			76.70%	86.30%
Diabetes			29.40%	31.60%
**Multi-site access audit**	2015		2017	
PD Catheters Inserted	1075		1131	769
Open	397		366	
Laparoscopic	196		232	
Surgical	593 (55%)		598 (49.0%)	325 (57.7%)
Medical	482 (45%)		533 (43.7%)	444 (42.2%)
Missing access type			90 (7.3%)	15 (1.9)

Supplemental methods

Instrument development, data collection and data sources

Survey instruments from PDOPPS were adapted for use in the UK and for this specific study. These included unit practice and medical director questionnaires that collected information at the facility, and the patient level, medical questionnaires (including investigator observed demographic data on age, sex, race and comorbidities), interval summaries, a study termination form, and a patient questionnaire. Instruments designed and collected specifically for the UK Catheter Study were 1) the catheter insertion worksheet; 2) the catheter event worksheet; 3) the catheter care and follow-up form; and 4) the patient training worksheets (event driven, available in Supplemental material). These were developed and piloted with a range of health care professionals and patients through focus groups, visits to several dialysis centres and with the study steering group. These study instruments describing catheter insertion and event management were completed by research staff at study sites and returned to the sponsor for data inputting. Completed paper PDOPPS patient questionnaires were returned to the UK sponsor before being shipped to Arbor Research for data inputting, while staff questionnaires on practice patterns and centre characteristics were completed by nursing and medical staff and submitted directly to Arbor Research. Time-varying patient data (e.g. peritonitis events, hospitalisation, laboratory data etc) was entered every four months by trained research staff into an electronic case report form (PDOPPSLink), supported by a national study coordinator. Patient-reported outcomes included health related quality of life (SF-12), assistance with PD and their experience of training and care every three months for the first 12 months.

Three dialysis unit-level measures, the percentage of patients starting PD within 1 year of kidney replacement therapy (KRT) initiation, the number of patients starting PD within a year of KRT initiation (both from a 4 year cohort), and the percentage of late starters, *i.e.* patients presenting to a nephrologist less than 90 days before KRT initiation, were extracted from the 2015 UK Renal Registry report.(12)

Supplementary Text 2: Supplemental sequences of regressions modelling

Sequences of regressions (1-4) a subclass of graphical models,(5) is a multivariate statistical model that extends path analysis (6) and provides a novel strategy for describing complex interrelations by jointly modelling multiple sets of outcomes variables and demographic factors.

The primary aim of the analysis was to describe the interrelationships between clinical history, and centre- level factors with catheter insertion related procedures and their impact on the occurrence of a catheter-related event within 12 months follow-up, separating direct from indirect associations via centre-level factors. The graphical model was specified by classifying the variables into sets of primary, intermediate, and demographic variables chosen to reflect their role in the study and ordered to reflect the likely direction of associations (Figure 1 - manuscript). The variables located on the left are responses to those located to their right, and variables collocated within boxes are symmetrically associated, therefore:

1. The occurrence of first catheter-related event contained in the first box on the left is a response variable to all variables positioned on its right (catheter insertion related procedures, dialysis unit level measures, patient clinical history and patient demographics).
2. The second block from left to right contains multiple response variables for catheter insertion related procedures which are response variables for all those positioned on its right (dialysis unit level measures, patient clinical history and patient demographics).
3. The next block contains multiple dialysis unit-level measures which are responses to patient clinical history and patient demographics.
4. Finally, patient clinical history measures are multiple response variables to patient demographics.

The model was built by fitting ordered sequences of logistic regression models for binary outcomes and linear regression models for continuous outcomes, for each variable in the different blocks of variables. Each variable in turn, starting with the occurrence of a catheter-related event and moving from left to right (Figure 1 - manuscript), was fitted into a regression model with all the variables to its right-hand side as explanatory. Patient-level variables were clustered within centre, a random intercept was added to their regression model to account for the correlation between observations from patients treated in the same centre. The regression models that best fitted the data were selected by comparing nested regression models with different combinations of explanatory variables using Wald tests.

The sequences of regressions model can also estimate the association between any two variables located within the same domain. We estimated this for the variables "number of patients starting PD within a year of KRT initiation", "proportion of patients starting PD within one year of KRT initiation" and "proportion of patients presenting to a nephrologist less than 90 days before KRT initiation" because these are closely related measures that were associated with the occurrence of a catheter event.

The sequences of regressions model is characterised by a regression graph with nodes representing variables arranged in blocks of response variables and demographic factors, connected by lines or arrow lines (Figure 2 – manuscript):

1. An arrow emerging from an explanatory variable and pointing to a response variable represents a *directed* association,

2. a sequence of connected arrow lines between two variables represents an *indirect* association, i.e., an association between two variables with intermediate explanatory variables, and
3. a dashed line connecting two variables depicts an *undirected* association.

The fitted model used partial regression coefficients to quantify the relative importance of the associations represented by the lines and arrow lines. In the fitted model, all the response variables which had at least one important explanatory variable were binary, therefore odds ratios (OR) and 95% intervals were reported. The presence or absence of an arrow line between two variables located in different boxes indicates whether they were associated or not after partitioning out the effects of all their combined explanatory variables. A sequence of connected arrow lines between 2 variables represents an indirect association. A dashed line between any two variables within a box represents symmetric associations. These were estimated for “number of patients starting PD within a year of KRT initiation”, “percentage of patients starting PD within one year of KRT initiation” and “percentage of late presenters” only. These associations were quantified with a partial correlation coefficient, or equivalently a Pearson correlation coefficient as these variables did not have explanatory variables in the fitted model. These variables are placed within a box in the regression graph while all other variables are presented in stacked boxes within their group category to make this distinction in Figure 2 of the manuscript.

Although the model fitting required multiple statistical tests, the components of the model reflect distinctive relations of interest, arising from the postulated ordering of the variables (Figure 1 - manuscript). Therefore, the interpretation of each significance level reported is valid, and adjustment for multiple testing is not required (5).

The graphical model used was designed to describe interrelations between multiple patient- centre-level factors underlying the occurrence of a catheter-related event in a single multivariate statistical model, rather than describing separate regression models for the single outcome variables where confounding between explanatory variables can hamper the understanding of the mechanism underlying the associations. Furthermore, the model estimation is straightforward and transparent because it relies on the estimation of local regression analyses. This provides a better insight into the interrelations and allows the identification of nonlinear relations and checks of model assumptions (7).

An expectation-maximisation (EM) algorithm for multivariate linear mixed-effects models for incomplete data was used for missing data imputation. An imputed dataset was used for model selection and multiple imputation based on 20 independent imputed datasets was carried out to estimate standard errors. Sensitivity analyses were carried out on all available data and complete-case data.

All models were fitted using maximum likelihood estimation. To check for non-linear terms, quadratic or interaction terms were included whenever these were highlighted by screening plots appropriate for checking the assumptions of the multivariate normal distribution. Diagnostic plots, including plots of residuals, and Q–Q plots were used to check the model assumptions. The goodness of fit was assessed through the model’s R-squared and the deviance statistics for continuous and discrete outcomes respectively.

Supplementary Text 3: **Supplemental multistate model methods**

The multistate model (Figure 3 – manuscript) analysed the patient event history, characterised by 6 states: catheter insertion, catheter-related event, PD, temporary or permanent transfer to HD, kidney transplant and death. Each patient had a sequence of transitions from one state to another coupled with the time (days from baseline) of transition. The figure shows the six states and 14 transitions allowed by the model. The states catheter insertion, catheter-related event, transfer to haemodialysis and PD had reversible transitions while kidney transplant and death were absorbing states. The primary parameters of interest were the hazard of transitions from catheter insertion to catheter related event and PD to catheter related event. A time-varying binary variable "catheter insertion technique" was created to indicate what insertion technique was used (medical or surgical) for a catheter that had an event.

The hazard functions of transitions from the states of catheter insertion and PD to catheter event were modelled in terms of catheter insertion technique and factors that were found to be directly associated with the probability of a catheter-related event within a year in the sequences of regressions model. The hazard functions for the transitions between the PD state and the states of kidney transplant and death included age, race, sex, and comorbidities as explanatory variables, with BMI added to the hazard functions for the transitions to death.

The model fitted each transition function separately by maximum likelihood estimation, assuming a Weibull distribution for the time to event and the semi-Markov property, whereby the hazard of moving from one state to another depends on the time since entry into the current state. Hazard functions were presented to visualise changes of transition rates over time and non-parametric estimates were included to assess the fit of the parametric models. Diagnostic tests of residuals were used to check the proportional hazards assumption.

References

(1) Wermuth N, Sadeghi K. Sequences of regressions and their independences. Test 2012;21(2):215-252.

(2) Wermuth N, Cox D. Concepts and a case study for a flexible class of graphical Markov models. In: Becker C, Fried R, Kuhn S, editors. Robustness and complex data structures: festschrift in honour of Ursula Gather. Germany: Springer 2013;331-350.

(3) Solis-Trapala I, Schoenmakers I, Goldberg GR, Prentice A, Ward KA. Sequences of regressions distinguish nonmechanical from mechanical associations between metabolic factors, body composition, and bone in healthy postmenopausal women. The Journal of Nutrition 2013;146:846-854.

(4) Lawson CA, Solis-Trapala I, Dahlstrom U, Mamas M, Jaarsma T, Kadam UT, et al. Comorbidity health pathways in heart failure patients: A sequences-of-regressions analysis using cross-sectional data from 10,575 patients in the Swedish Heart Failure Registry. PLoS Med 2018;15 (3): e1002540. https://doi.org/10.1371/journal.pmed.1002540

(5) Cox D, Wermuth N. Multivariate Dependencies: Models, Analysis and Interpretation. Oxford: Chapman & Hall/CRC;1996.

(6) Wright S. The Method of Path Coefficients. 1934;:161-215.

(7) Hardt J, Petrak F, Filipas D, Egle UT. Adaptation to life after surgical removal of the bladder-an application of graphical Markov models for analysing longitudinal data. Stat Med 2004;23(4):649-666.

Supplementary Text 4: Supplemental interpretation of results from the sequences of regressions modelling

This section completes the interpretation of the sequence of regression models developed for each outcome variable.

Factors influencing catheter-related events within 1 year

The associations between centre-level factors and catheter events described in the Results Section of the manuscript can be illustrated by comparing the odds of a catheter event for patients in centres with factors' values equal to the first and the third quartiles of the distribution of the variable we are reporting, respectively. The odds of a catheter event increased by a factor of 1.5 for patients in a centre in which 18% compared to 31% of its patients started PD within one year of commencing KRT. Likewise, the odds of a catheter event increased by a factor of 1.3 for 18% compared to 14% of a centre's patients being late presenters. The percentage of patients starting PD within one year of KRT initiation within a centre was correlated with the total number of patients starting PD and with the percentage of late presenters (partial correlation coefficients 0.22 and 0.24 respectively).

Catheter insertion related procedures

For two identical patients, the one treated in a centre averaging 12.3 patients starting PD per year (lower quartile) compared with 39.8 (upper quartile), the odds of a medical insertion was estimated to be increased by a factor of 25.9 for the latter. Likewise, compared to patients without the diagnosis, the odds of a surgical insertion for patients with diagnosed cystic, hereditary, or congenital disease were 8.3-fold greater, and for patients with a history of abdominal, genitourinary, or gastrointestinal surgery were 3.6-fold greater.

PD subtype (CAPD *vs* APD) was directly explained by percentage of late starters in centre (OR 1.25, 95% CI 1.01 to 1.53), history of abdominal, genitourinary, or gastrointestinal surgery (OR 1.76, 95% CI 1.03 to 3.02), sex (OR 1.65, 95% CI 1.03 to 2.63) and indirectly by the effect of age and sex on history of abdominal genitourinary or gastrointestinal surgery. Deep cuff position (paramedian *vs* central or midline) was directly explained by BMI (OR 1.07, 95% CI 1.10 to 1.13). Patient saw nephrologist (more than 6 months *vs* less than 6 months) was directly explained by diagnosis of cystic, hereditary, or congenital disease (OR 2.23, 95% CI 0.98 to 5.05), age (OR 1.01, 95% CI 1.00 to 1.03), and BMI (OR 1.05, 95% CI 1.00 to 1.10).

Dialysis unit level measures

Being at a transplant unit was directly associated with history of abdominal, genitourinary, or gastrointestinal surgery (OR 1.65, 95% CI 1.12 to 2.42) and younger age (OR 0.99 95% CI 0.98 to 1.00). longer duration of training (more than 4 days *vs* 2-3 days) was directly associated with age (OR 0.99, 95% CI 0.8 to 1.00).

The PD facility being affiliated with a university was directly associated with sex (OR 0.68, 95% CI 0.50 to 0.92), age and race, with an interaction between the latter. The odds of being in a university affiliated PD facility were estimated to be reduced by a factor of 0.64 for a white patient compared to a non-white patient if they were both 47 years old (the lower quartile), and by a factor of 0.24 if they were 71 years old (the upper quartile). The number of patients starting dialysis per year, the

percentages of patients starting PD within 1 year of RRT initiation and late starters in centre did not have explanatory variables. Logistic regression analyses for the remaining centre based variables are presented in Supplemental Table 2 and Supplemental Figure 5.

Patient clinical history

Supplemental Table 5 and Supplemental Figure 4 show associations between sex, age, BMI and race with key co-morbidities, primary renal diagnoses, surgical history and catheter insertion. Importantly diabetes, lung disease, peripheral vascular disease and coronary artery disease were less common in women whereas history of genitourinary surgery was more common. There was an interaction between age and sex on the latter. The odds of a history of genitourinary surgery were estimated to be 2.4-fold greater for a female than a male participant if they were both 47 years old (the lower quartile), and 1.2-fold greater if they were 71 years old (the upper quartile). Age was associated with lung disease, PVD, coronary artery disease and history of genitourinary surgery but negatively with cystic, hereditary of congenital disease. BMI was positively associated with diabetes and negatively with history of cystic, hereditary of congenital disease.

Supplemental figures

Supplemental Figure 1 CONSORT diagram. * Estimated from registry returns

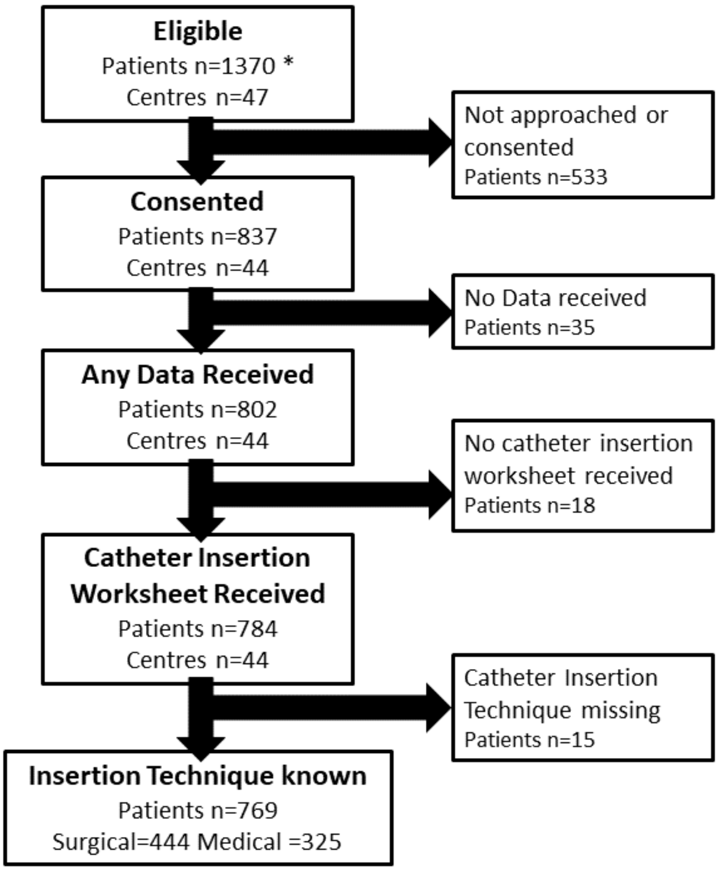

Supplemental Figure 2 External generalizabilty of PD catheter insertion techniques – Comparison with the UK Renal Registry
Blue: Surgical Insertion Technique, Yellow: Medical Insertion Technique, Green: Unknown Insertion Technique.

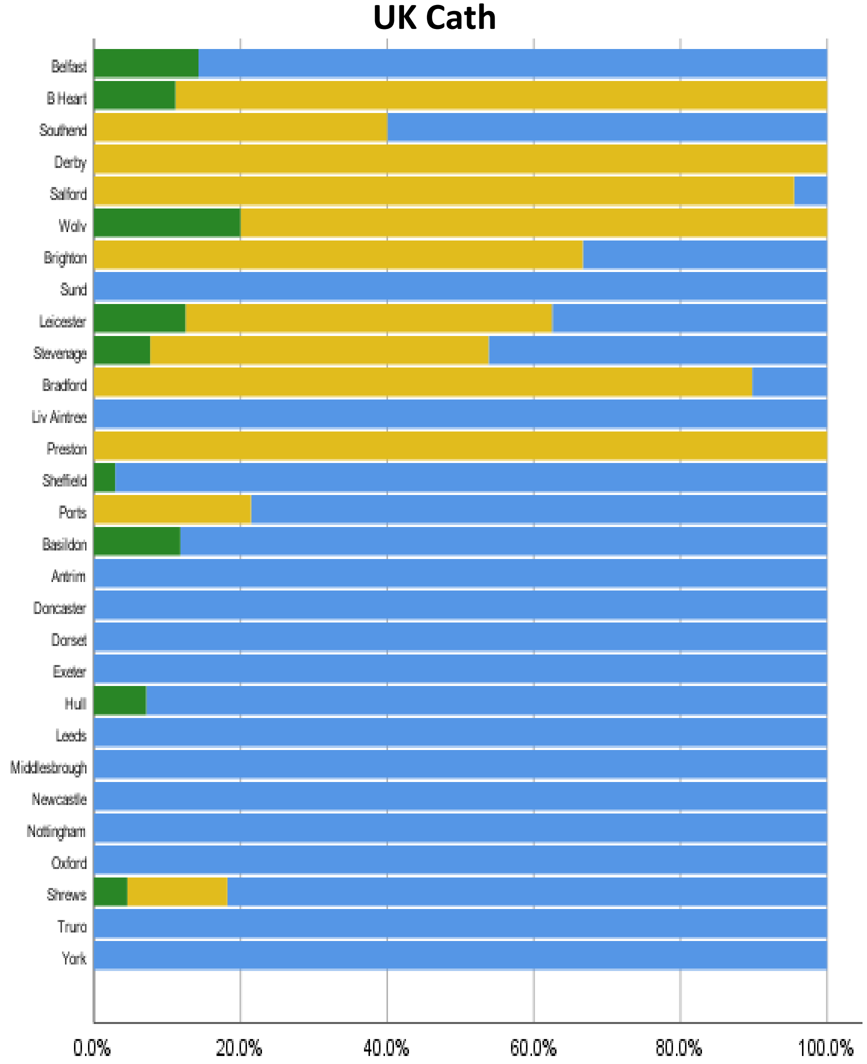

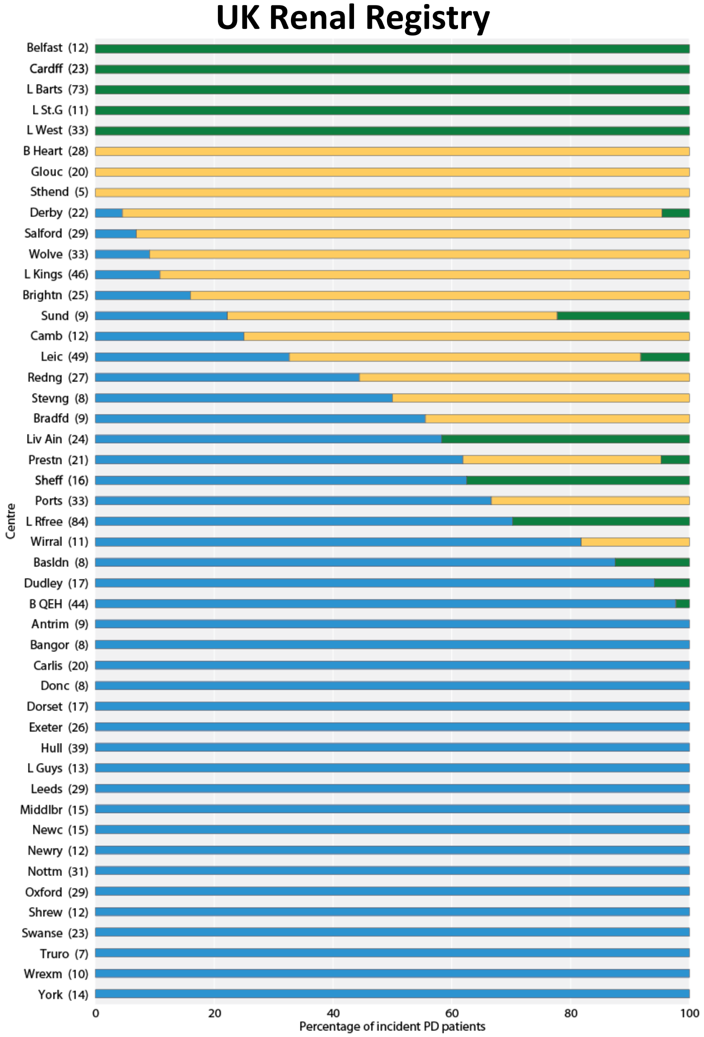

Adapted from Nephron 2017;137(suppl1):269–296
DOI: 10.1159/000481374

Supplemental Figure 3 Regression subgraph for catheter-related event as response variable. An arrow line indicates a direct association with catheter event, the strength of this association is shown as OR (95% CI). A dashed line indicates an important undirected association between a pair of variables characterising the size of centre, percentage of patients on PD within 1 year of RRT initiation and percentage of late presenters measured by a correlation coefficient (95% CI).

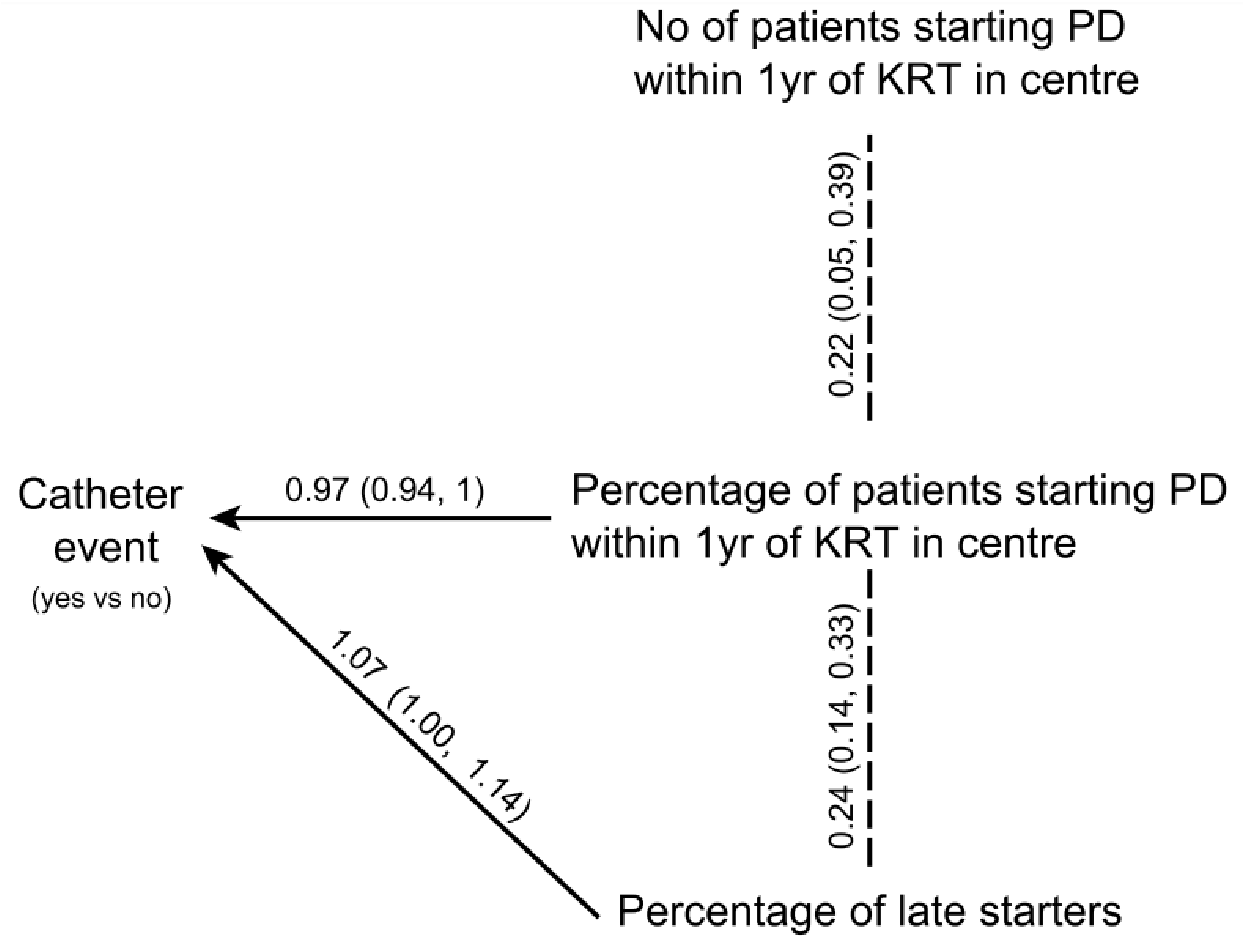

Supplemental Figure 4 Regression subgraph for catheter insertion related procedures. An arrow line emerging from an explanatory variable and pointing to a response variable of the same colour represents a direct association, controlling for all remaining regressors. The strength of this association is shown as OR (95% CI), categories of binary variables are indicated in parenthesis to aid interpretation of odds ratios. A dashed line was used to indicate a significant undirected association quantified by a Pearson correlation coefficient between a pair of variables characterising the number of patients starting PD within 1 year of RRT, percentage of patients starting PD within 1 year of RRT and percentage of late presenters in centre. These variables are placed within a box in the graph while all other variables are presented in stacked boxes within their group categories as undirected associations were not estimated for the latter. A significant interaction of age by gender on history of genitourinary surgery is also indicated and shown on Supplemental Table 3.

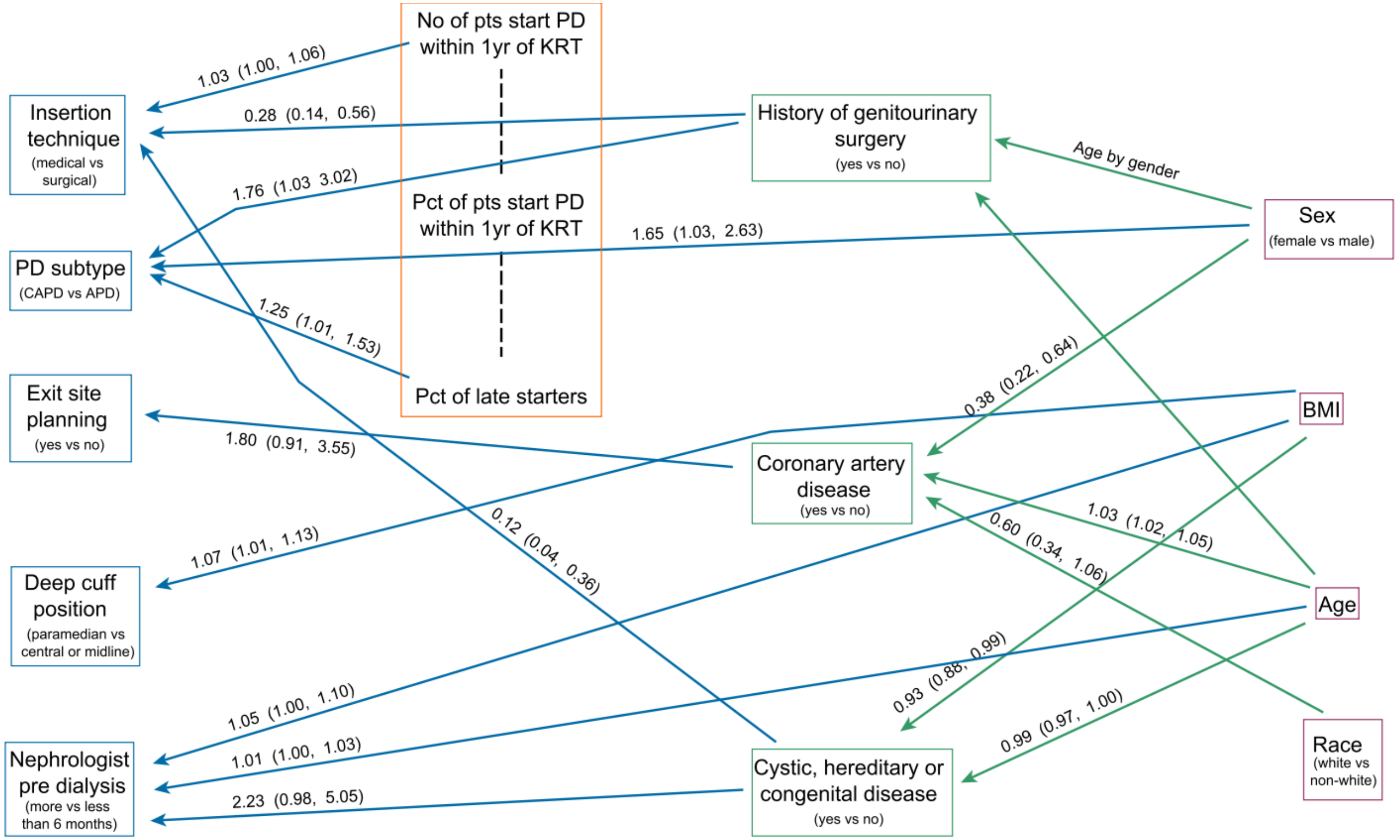

Supplemental Figure 5 Regression subgraph for dialysis unit measures and patient clinical history. An arrow line emerging from an explanatory variable and pointing to a response variable of the same colour represents a direct association, controlling for all remaining regressors. The strength of this association is shown as OR (95% CI), categories of binary variables are indicated in parenthesis to aid interpretation of odds ratios. Significant interactions are also indicated and shown on Supplemental Tables 2 and 3.

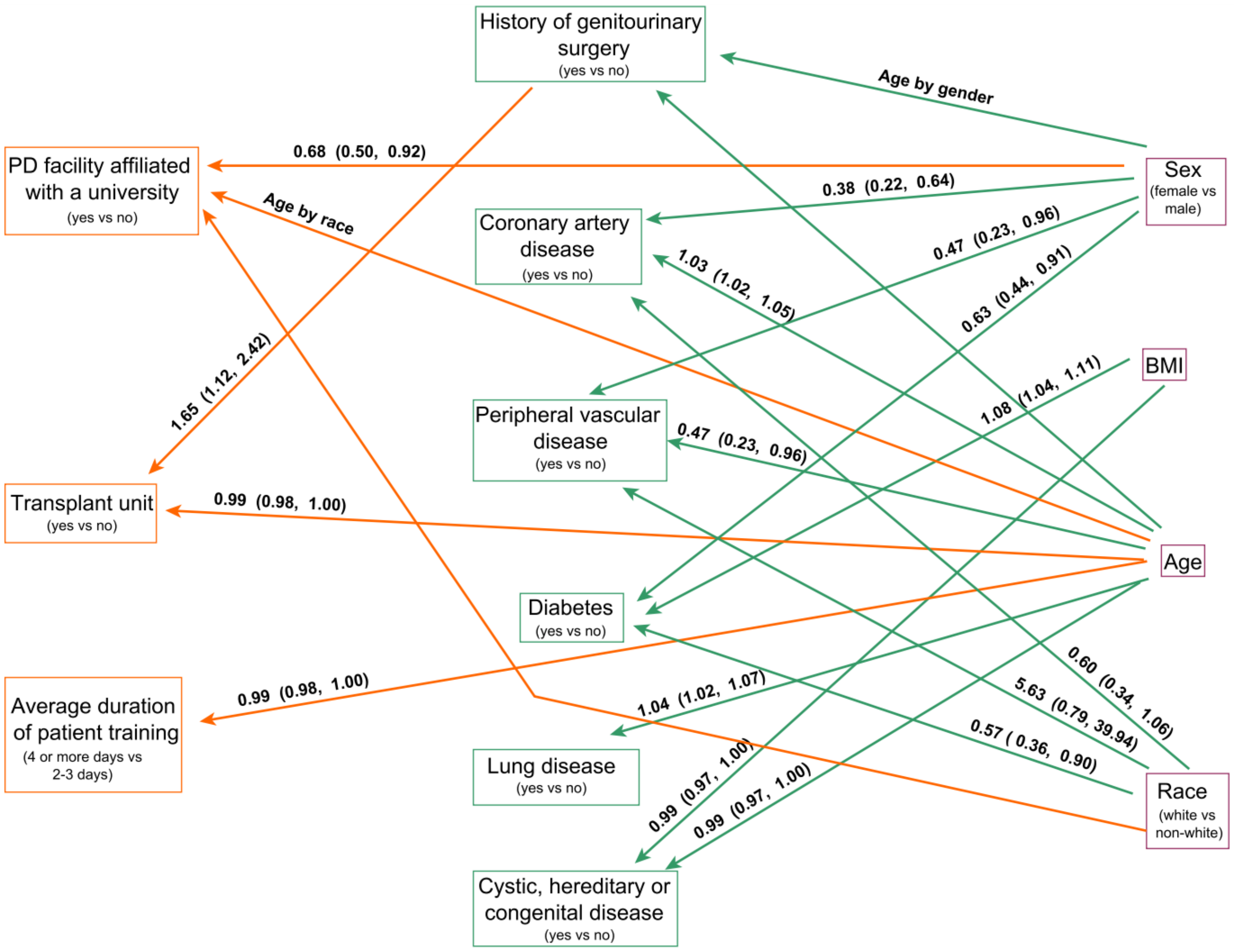

Supplemental Figure 6 Cumulative hazard functions of selected multistate transitions. Model-based estimates and their 95% confidence bands are shown in blue full and dashed lines respectively, non-parametric estimates reflecting goodness of fit and their 95% confidence bands are shown in black full and dashed lines.

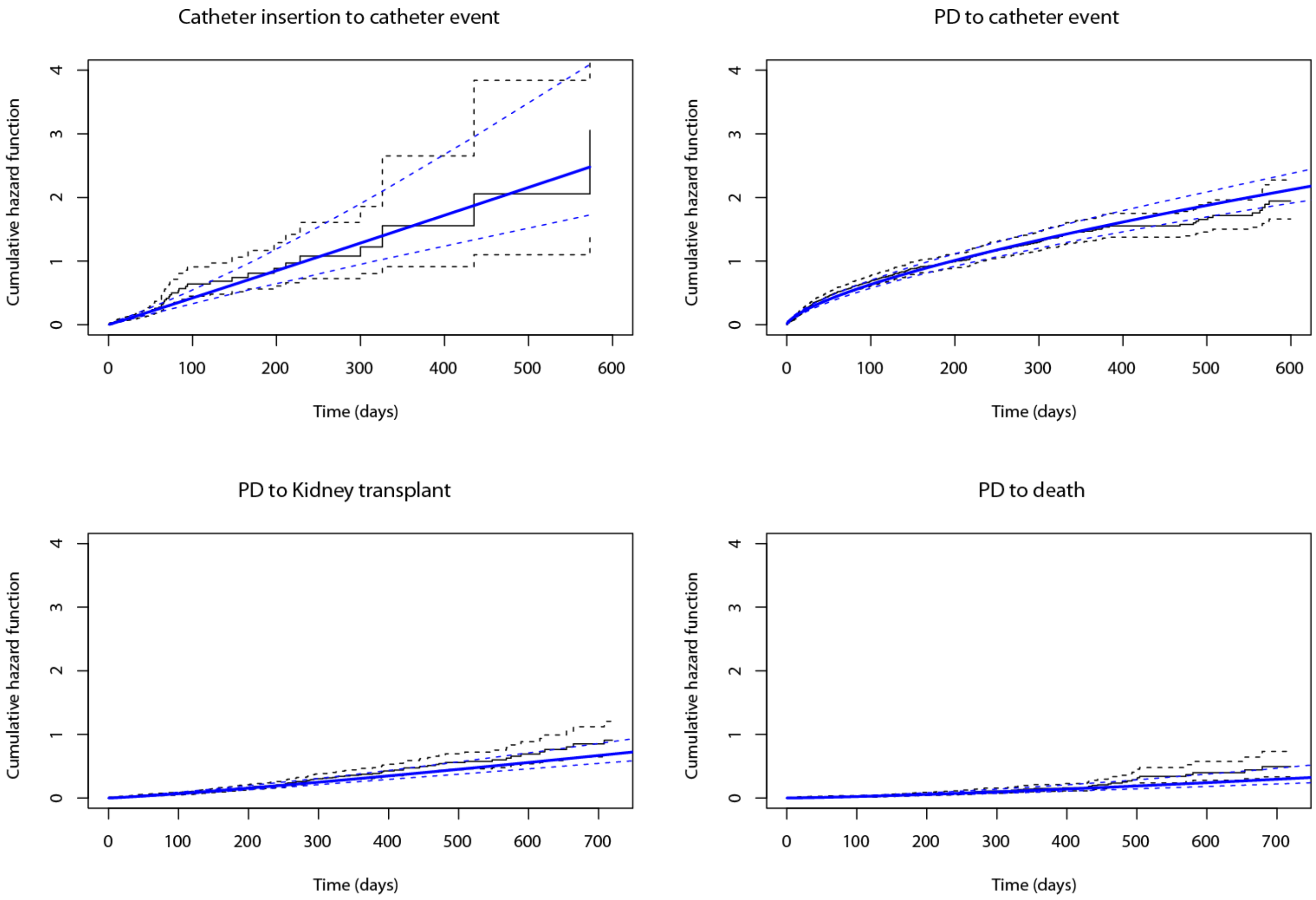

Supplemental Tables

Supplemental Table 1 – Description of variables used in the initial sequences of regressions model (Figure 1)

Variable description
Participant's first catheter-related event during first year in study (1= first catheter-related event, 0=no events occurred), where catheter-related event is a composite outcome with one or more of the following causes: catheter function, peritonitis, exit site infection or hospitalisation
Catheter insertion technique (1=medical, 0=surgical)
Was there formal documentation of exit site planning with the patient prior to catheter insertion? (1=yes, 0=no)
Deep cuff position (1=paramedian, 0=central or midline)
Did the patient receive nasal mupirocin prior to the procedure? (1=yes, 0=no)
How many months before the start of chronic dialysis did the patient first see a nephrologist? (1= more than 6 months, 0=less than 6 months)
PD Subtype in use on listing date (or planned subtype for first home exchange) (1=CAPD, 0=APD)
How often does your program review rates of PD-related infections for the purposes of continuous quality improvement? (1=Every 1-3 months, 2=Every 3-6 months, 3=Every 6-12 months, 4= Every 1-2 years, 5= Never)
What is the average duration of patient training prior to PD initiation? (1= 4 or more days, 0= 2-3 days)
What best describes the affiliation of the nurses who initially train patients? (1= all nurses employed at the facility, 0= combination of nurses employed at the facility and third-party nurses)
Can patients at your facility contact a PD nurse by phone after regular working hours? (1=yes, 0=no)
Transplant unit (1=yes, 0=no)
Is your PD facility affiliated with a university? (1=yes, 0=no)
Percentage of Patients presenting <90 days before RRT initiation in centre as per UK Renal Registry data
Estimated percentage of patients starting PD within one year of starting dialysis in centre, as per UK Renal Registry data
Number of patients starting PD in a 4-year period in centre as per UK Renal Registry data
History of diabetes (1=yes, 0=no)
History of lung disease (1=yes, 0=no)
History of peripheral vascular disease (1=yes, 0=no)
History of congestive heart failure (1=yes, 0=no)
History of coronary artery disease (1=yes, 0=no)
Glomerulonephritis is the primary cause of end stage renal disease (1=yes, 0=no)
Cystic, hereditary, or congenital disease is the primary cause of end stage renal disease (1=yes, 0=no)
Prior to the initiation of peritoneal dialysis, has the patient had a previous history of abdominal, genitourinary, or gastrointestinal surgery (not including kidney transplants)? (1=yes, 0=no)
Sex (1=female, 0=male)
Age in years
Body mass index (kg/m2)
Race (1=white, 0=other)

Supplemental Table 2 – Primary hospitalisation codes for catheter events purely relating to hospitalisation. As diagnoses and procedures can trigger hospitalisations therefore leading to multiple codes per admission, the first diagnosis then if absent the first procedure is reported below.

Code	Description	Medical	Surgical
D159	Constipation	1	6
D200	Peritoneal dialysis associated peritonitis	3	9
D201	Peritoneal dialysis associated exit site infection	2	2
D202	Peritoneal dialysis catheter leak	1	4
D203	Peritoneal dialysis catheter complication (other)	3	4
D204	Peritoneal dialysis related hernia	1	3
D205	Hemoperitoneum	0	1
D211	Peritoneal dialysis catheter malfunction	25	23
P110	Peritoneal dialysis catheter revision/repositioning	1	1
P111	Peritoneal dialysis catheter insertion	1	4
P112	Peritoneal dialysis catheter removal	2	2
	Total	40	59

Supplemental Table 3 - Models describing direct associations for catheter-related event and catheter insertion procedures as response variables (Supplemental Figure 4). The models were built by fitting ordered sequences of logistic regressions with a random intercept to each variable, including all the variables to their right in the postulated model (Figure 1) as explanatory variables. For each outcome, the regression model that best described the data was selected by comparing nested models with different combinations of explanatory variables.

Dependent variables (outcome for each regression model)

		Catheter insertion related procedures				
**Explanatory variable**	**Catheter-related event, yes vs no OR (95% CI)**	Insertion technique, medical vs surgical OR (95% CI)	PD subtype, CAPD vs APD OR (95% CI)	Exit site planning, yes vs no OR (95% CI)	Deep cuff position, paramedian vs central or midline OR (95% CI)	Patient saw nephrologist, more vs less than 6 months OR (95% CI)
**Dialysis unit level measures**						
Proportion of late presenters	1.07 (1.00, 1.14)		1.25 (1.01, 1.53)			
Proportion of patients starting PD within 1 year of KRT	0.97 (0.94, 1.00)					
No of patients starting PD within 1 year of RRT		1.03 (1.00, 1.06)				
**Patient clinical history**						
Comorbid conditions						
Coronary artery disease, yes				1.80 (0.91, 3.55)		
Primary cause of end stage renal disease						
Cystic, hereditary or congenital disease, yes		0.12 (0.04, 0.36)				2.23 (0.98, 5.05)
History of abdominal, genitourinary, or gastrointestinal surgery, yes		0.28 (0.14, 0.56)	1.76 (1.03, 3.02)			
**Patient demographics**						
Sex, female			1.65 (1.03, 2.63)			
Age in years						1.01 (1.00, 1.03)
Body mass index, kg/m2					1.07 (1.01, 1.13)	1.05 (1.00, 1.10)

Supplemental Table 4 - Models describing direct associations for dialysis unit level measures as response variables (Supplemental Figure 5). The models were built by fitting ordered sequences of logistic regressions to each variable, including all the variables to their right in the postulated model (Figure 1). For each outcome, the regression model that best described the data was selected by comparing nested models with different combinations of explanatory variables. In addition to the direct associations shown in the table, there was a correlation (95% CI) of 0.24 (0.14 to 0.33) between proportion of patients starting PD within 1 year of KRT and proportion of late presenters in centre. Number of patients starting PD within 1 year of KRT was correlated with percentage of patients starting PD within 1 year of KRT (correlation 0.22, 95% CI 0.05 to 0.39).

	**Dependent variables (outcome for each regression model)**		
	**Dialysis unit level measures**		
**Explanatory variables**	Average duration training, 4 or more days vs 2-3 days, OR (95% CI)	Transplant unit, yes vs no, OR (95% CI)	PD facility affiliated with a university, yes vs no, OR (95% CI)
**Patient clinical history**			
Comorbid conditions			
History of abdominal, genitourinary, or gastrointestinal surgery, yes		1.65 (1.12, 2.42)	
**Interaction terms**			
age (per year) and white			0.96 (0.94, 0.99)
**Patient demographics**			
Sex, female			0.68 (0.50, 0.92)
Age	0.99 (0.98, 1.00)	0.99 (0.98, 1.00)	1.03 (1.00, 1.05)
Body mass index, kg/m2			
Race			
White			4.33 (0.85 21.95)
Other			Reference

Supplemental Table 5 - Models describing direct associations for clinical history as response variables (Supplemental Figure 5). The models were built by fitting ordered sequences of logistic regressions with a random intercept to each variable, including all the variables to their right in the postulated model (Figure 1), *i.e.,* patient demographics. For each outcome, the regression model that best described the data was selected by comparing nested models with different combinations of explanatory variables.

	**Dependent variables (outcome for each regression model)**					
	**Patient clinical history**					
**Explanatory variables**	Diabetes, yes vs no OR (95% CI)	Lung disease, yes vs no OR (95% CI)	Peripheral vascular disease, yes vs no OR (95% CI)	Coronary artery disease, yes vs no OR (95% CI)	Cystic, hereditary or congenital disease, yes vs no OR (95% CI)	History of genitourinary surgery, yes vs no OR (95% CI)
**Patient demographics**						
Sex, female	0.63 (0.44, 0.91)		0.47 (0.23, 0.96)	0.38 (0.22, 0.64)		10.14 (2.08, 49.44)
Age		1.04 (1.02, 1.07)	1.02 (1.00, 1.04)	1.03 (1.02, 1.05)	0.99 (0.97, 1.00)	1.04 (1.02, 1.05)
Body mass index, kg/m2	1.08 (1.04, 1.11)				0.93 (0.88, 0.99)	
Race						
White	0.57 (0.36, 0.90)		5.63 (0.79, 39.94)	0.60 (0.34, 1.06)		
Other	Reference			Reference		
**Interaction term**						
age (per year) and female						0.97 (0.95, 1.00)

Supplemental Table 6 – Table of observed transitions described in Figure 3.

**Frequency of observed transitions: number of times each pair of states was observed at successive observation times**						
	**to**					
**from**	Catheter Insertion	Catheter Event	Peritoneal Dialysis	Haemodialysis	Kidney Transplant	Death
Catheter Insertion	13	201	595	0	0	0
Catheter Event	20	23	560	156	0	0
Peritoneal Dialysis	33	673	58	99	154	67
Haemodialysis	14	58	22	0	0	17
Kidney Transplant	0	0	0	0	0	0
Death	0	0	0	0	0	0

PDOPPS
Peritoneal Dialysis Outcomes and Practice Patterns Study

Catheter Event Worksheet
UK Catheter Study

Patient Study ID:
Centre ID:

This form is to be completed at the time of each procedure that impacts on peritoneal access.

INSTRUCTIONS FOR FILLING IN THIS FORM – Mark an 'X' in the appropriate box(es).

A	PATIENT STATUS	
A-1	Date of Access Procedure	___/___/_____<br>DD MM YYYY
A-2	Indication for the procedure (event code)<br>☐ Dialysis Fluid Leak Pericatheter<br>☐ Dialysis Fluid Leak, Not Pericatheter<br>☐ Hernia<br>☐ Poor or Absence of Inflow/Outflow<br>☐ Catheter Displacement/Fell Out<br>☐ Exit Site Infection	☐ Tunnel Infection<br>☐ Wound Infection<br>☐ Peritonitis<br>☐ Bleeding (from wound, exit site or intraperitoneal)<br>☐ Catheter-Related Pain<br>☐ Other:<br>Please specify………………………………………………
A-3	Procedure code<br>☐ Repositioning Surgical/Laparoscopic<br>☐ Repositioning Non-Surgical and/or Fluoroscopic<br>☐ Surgical Procedure<br>☐ With No Additional Procedure<br>☐ With Hernia Repair<br>☐ With Omentopexy<br>☐ With Omentectomy<br>☐ With Other Procedure (Please specify procedure name. If unknown, specify unknown.)<br>☐ Unknown if Additional Procedure<br>☐ Removal of Catheter<br>☐ Removal of Catheter with Replacement During the Same Procedure<br>☐ Exit Site/Tunnel Revision<br>☐ Cuff Shaving<br>☐ Intracatheter Thrombolytic Agent (i.e., Urokinase, Tissue Plasminogen Activator [TPA])<br>☐ PD Catheter Exteriorization	

	☐ Replacement with a new catheter<br>☐ Other: Please specify ……………………………………………………………
A-4	Was a laparoscope used during this procedure Y☐ N ☐

**B**	**OPERATOR FACTORS**	
B-1	a. Name of operator responsible for the procedure	
	b. Was the operator present for the entire case?	Y☐ N ☐
B-2	Did a trainee perform the procedure?	Y☐ N ☐
B-3	Who Inserted the catheter?<br>☐ Surgeon<br>☐ Nephrologist<br>☐ Nurse Practitioner<br>☐ Radiologist<br>☐ Other: Please specify………………………………………………………………………	
B-4	How many PD catheter insertions has this operator performed in the last 12 months?	

**C**	**PRE-INSERTION CARE**	
C-1	Did the patient receive pre-operative antibiotics?	Y☐ N ☐
C-2	What was the route of admin?<br>☐ Intravenous<br>☐ Oral<br>☐ Other: Please specify ………………………………………………………………………	
C-3	Was the antibiotic a…<br>☐ Cephalosporin<br>☐ Vancomycin<br>☐ Other: Please specify …………………………………………………………………………	
C-6	What type of anesthesia was used?<br>☐ local anaesthetic<br>☐ Spinal anaesthetic<br>☐ general anaesthetic<br>☐ Local anaesthetic + Sedation<br>☐ Other: Please specify…………………………………………………………………….…	

**D**	**INSERTION CARE**
D-1	Where did the procedure take place?<br>☐ Operating theatre<br>☐ X-ray department<br>☐ Ward Treatment Room<br>☐ Other: Please<br>Specify…………………………………………………………………..…

E	POST-INSERTION CARE	
E-2	Was this intended to be a day case catheter insertion?	Y☐ N☐ Unknown ☐
E-3	Was the patient discharged home the same day as the catheter insertion?	Y☐ N☐ Unknown ☐
E-4	Destination of patient after the procedure<br>☐ Renal Specialist Ward<br>☐ Non-Specialist Medical Ward<br>☐ Non-Specialist Surgical Ward<br>☐ Day Case Unit<br>☐ Other: Please specify<br>………………………………………………………………………	
E-5	Was the catheter insertion attempt successful at the point of discharge?	Y☐ N☐ Unknown ☐

Date this form completed ....

Signature of person completing this form ....

Print name………………………………………………………………………………………

PDOPPS
Peritoneal Dialysis Outcomes and Practice Patterns Study

UK cath

Catheter Insertion Worksheet
UK Catheter Study

Patient Study ID:

Centre ID:

This form is to be completed at the time of each peritoneal catheter insertion.

INSTRUCTIONS FOR FILLING IN THIS FORM – Mark an 'X' in the appropriate box(es).

**A**	**PATIENT STATUS**	
A-1	Date of Access Procedure	___/___/_____ DD MM YYYY
A-2	Weight (if not measured, ask the patient) __________________	______________________ 0: kg 1:lbs
A-3	Height (if not measured, ask the patient) __________________	______________________ 1: cm 2: inches
A-4	Is this the patient's first, second or subsequent catheter?<br>☐ First<br>☐ Second<br>☐ Subsequent	
A-5	Was there formal documentation of exit site planning with the patient prior to catheter insertion?	Y☐ N☐ Unknown☐
A-6	Does the patient have abdominal scar(s)?	Y☐ N☐ Unknown☐

**B**	**OPERATOR FACTORS**	
B-1	a. Name of operator responsible for the procedure	
	b. Was the operator present for the entire case?	Y☐ N ☐
B-2	Did a trainee perform the procedure?	Y☐ N ☐
B-3	Who Inserted the catheter?<br>☐ Surgeon<br>☐ Nephrologist<br>☐ Nurse Practitioner<br>☐ Radiologist<br>☐ Other: Please specify……………………………………………………………………	
B-4	Approximately how many PD catheter insertions has this operator performed in the last 12 months?	5 or less ☐; 6 – 10 ☐<br>11 – 15 ☐; 16 – 20 ☐<br>More than 20 ☐

Tear here. *Fill in name for local identification only. Remove this section before returning this form to the Coordinating Centre.* Tear her

Patient's Name:

C	PRE-INSERTION CARE	
C-1	Did the patient receive pre-operative antibiotics?	Y☐ N ☐
C-2	What was the route of admin?<br>☐ Intravenous<br>☐ Oral<br>☐ Other: Please specify …………………………………………………………………………	
C-3	Was the antibiotic a…<br>☐ Cephalosporin<br>☐ Vancomycin<br>☐ Other: Please specify ……………………………………………………………………………	
C-4	Did the patient receive nasal antiseptic/antibiotic prior to the procedure?<br>☐ None<br>☐ Mupirocin<br>☐ Nasepin<br>☐ Gentamicin<br>☐ Other: Please specify……………………………	
C-5	Did the patient receive specific pre-procedure bowel preparation with a laxative?	Y☐ N ☐
C-6	What type of anesthesia was used?<br>☐ Local anaesthetic<br>☐ Spinal anaesthetic<br>☐ General anaesthetic<br>☐ Local anaesthetic + Sedation<br>☐ Other: Please specify……………………………………………………………………..…	

D	INSERTION CARE
D-1	Where did the procedure take place?<br>☐ Operating theatre<br>☐ X-ray department<br>☐ Ward Treatment Room<br>☐ Other: Please<br>Specify………………………………………………………………………..…
D-2	Tick the insertion technique below…<br>☐ Open Surgery (includes minilap)<br>☐ Laparoscopic Surgery<br>☐ Percutaneous (Non-Fluoroscopic-Assisted)<br>☐ Percutaneous (Fluoroscopic-Assisted)<br>☐ Peritoneoscopic<br>☐ Unknown

D-3	Antibacterial coating to catheter?	Y☐ N☐<br>Unknown☐
D-4	Catheter subtype – intraperitoneal segment<br>☐ Coiled Tip<br>☐ Straight Tip<br>☐ Unknown<br>☐ Other: Please<br>specify………………………………………………………………….…	
D-5	Catheter subtype – subcutaneous segment – circle below<br>☐ Swan Neck<br>☐ Straight<br>☐ Unknown<br>☐ Other: Please<br>specify………………………………………………………………….…	
D-6	Was this the intended catheter type?	Y☐ N ☐
D-7	If No, was the difference in catheter…<br>☐ Type<br>☐ Length<br>☐ Other: Please<br>specify………………………………………………………………….	
D-8	Number of cuffs<br>☐ 1 ☐ 3<br>☐ 2 ☐ Unknown	
D-9	Deep cuff position<br>☐ Paramedian ☐ Central / Midline	
D-10	Was the catheter length specifically selected according to the patient size?	Y☐ N☐<br>Unknown☐
D-11	Was the exit site location<br>☐ Presternal<br>☐ Upper Abdominal<br>☐ Lower Abdominal<br>☐ Other: Please<br>Specify……………………………………………………………….…	

D-12	Was an addition procedure undertaken at the time of catheter insertion?<br>☐ No additional procedure<br>☐ With Hernia Repair<br>☐ With Omentectomy<br>☐ With Omentopexy<br>☐ With Other Procedure (please specify. If unknown, please specify unknown)<br>☐ Unknown if Additional Procedure<br>☐ Other: Please Specify……………………………………………………………….…	
D-13	Was the catheter flushed post procedure?	Y☐ N☐<br>Unknown☐
D-14	Type of flushing fluid used<br>☐ Dialysate<br>☐ Normal Saline<br>☐ Other: Please Specify……………………………………………………………….…	
D-15	Was the final position of the catheter tip confirmed visually or by imaging at the end of the procedure?	Y☐ N☐<br>Unknown☐
D-16	Was the external portion of the catheter immobilized post procedure (eg using tape)?	Y☐ N☐<br>Unknown☐
D-17	Was a dressing applied to the exit site?	Y☐ N☐<br>Unknown☐

**E**	**POST-INSERTION CARE**
E-1	What type of access procedure was performed?<br>☐ PD Catheter – Initially Embedded<br>☐ PD Catheter – Never Embedded<br>☐ PD Catheter – Previous Embedded Status Unknown<br>☐ Native AV Fistula<br>☐ AV Graft<br>☐ Cuffed Hemodialysis Catheter (e.g. PermCath)<br>☐ Uncuffed Hemodialysis Catheter

E-2	Was this intended to be a day case catheter insertion?	Y☐ N☐ Unknown ☐
E-3	Was the patient discharged home the same day as the catheter insertion?	Y☐ N☐ Unknown ☐
E-4	Destination of patient after the procedure<br>☐ Renal Specialist Ward<br>☐ Non-Specialist Medical Ward<br>☐ Non-Specialist Surgical Ward<br>☐ Day Case Unit<br>☐ Other: Please specify<br>………………………………………………………………………	
E-5	Was the catheter insertion attempt successful at the point of discharge?	Y☐ N☐ Unknown ☐

Date this form completed ....

Signature of person completing this form ....

Print name……………………………………………………………………………………………

Catheter care and follow-up form

PDOPPS
Peritoneal Dialysis Outcomes and Practice Patterns Study

UK cath

Catheter Care and Follow up Form
UK Catheter Study

Patient Study ID:

Centre ID:

If the patient has had a peritonitis or exit site infection in the last 3 months please download and complete the infection worksheet from the UK Catheter Study website http://www.keele.ac.uk/uk-pdopps/

INSTRUCTIONS FOR FILLING IN THIS FORM – Mark an 'X' in the appropriate box(es).

A	CATHETER CARE AND FOLLOW UP	
	Date completed	___/___/______ DD MM YYYY
A-1	If the patient has been hospitalized in the last 3 months please insert the dates… (Hospitalization codes are available at the Study Website)	

Date Admitted	Date Discharged	Enter Code:	Diagnoses			Procedures		
DD/MM/YYYY	DD/MM/YYYY	1: Outpatient<br>2: Hospitalized<br>3: Admitted for Observation	1	2	3	1	2	3

A-2	Did this patient receive a catheter?	Y☐ N ☐
A-3	Date catheter inserted	___/___/______ DD MM YYYY
A-4	If the patient did not receive a catheter – is catheter insertion still planned?	Y☐ N ☐
A-5	If the patient did not receive a catheter – please select a reason from the following list (tick all that apply)<br>☐ dialysis not indicated yet<br>☐ patient medically unfit for catheter insertion<br>☐ patient required emergency haemodialysis<br>☐ patient has chosen an alternative modality	

	☐ Renal transplant (date of transplant) – complete study Termination Form<br>☐ patient has chosen not to have dialysis<br>☐ Logistical or organizational reason	
A-6	Does the patient currently receive renal replacement therapy? (If YES go to A-7; if NO go to A-8)	Y☐ N ☐
A-7	What type of renal replacement therapy is the patient receiving?<br>☐ Haemodialysis in centre (go to A-9)<br>☐ Haemodialysis at home – complete study Termination Form<br>☐ Peritoneal dialysis in centre (intermittent PD) (go to A-10)<br>☐ Renal transplant (date of transplant) – complete study Termination Form<br>☐ Peritoneal dialysis at home – complete Interval Summary Form	
A-8	What is the reason that the patient does not receive renal replacement therapy?<br>☐ Dialysis not indicated at this stage (level for renal function does not currently require it)<br>☐ Patient or family have chosen not to have dialysis – complete study Termination Form<br>☐ PD catheter does not work or has had a complication – insert reason code from the attached list here ☐<br>☐ PD catheter insertion has been delayed or rescheduled – please complete question A-4 & A-5 above.<br>☐ Patient has died – complete Death Detail Form	
A-9	Reason for in centre haemodialysis – insert reason code from the attached list here (most important first)<br>☐ 1. _____________<br>☐ 2. _____________<br>☐ 3. _____________	
A-10	Reason for intermittent in-centre peritoneal dialysis (tick most relevant)<br>☐ Patient unable to train to perform PD due to cognitive reason<br>☐ Patient unable to train to perform PD due to physical reason<br>☐ Patient unable to dialyse at home due to problems with accommodation<br>☐ Patient unable to dialyse at home due to social circumstances<br>☐ Patient has not trained yet due to organizational reasons<br>☐ Patient has not yet trained yet because of insufficient time since catheter insertion for training to have occurred	

Reason for Departure including Transfer to or Addition of Hemodialysis		
INFECTION-RELATED<br>1. Peritonitis<br>1a. Acute Severe<br>1b. Refractory<br>1c. Relapsing<br>1d. Recurrent<br>2. Exit Site Infection<br>2a. Exit Site without tunnel infection<br>2b. Tunnel Infection<br><br>CATHETER-RELATED PROBLEMS<br>3. Catheter Blockage<br>3a. Fibrin<br>3b. Omental Wrap<br>3c. Adhesions<br>3d. Catheter Misplaced<br>3e. Cause Unclear<br>4. Catheter Displacement<br>4a. Cuff Extrusion<br>4b. Catheter Fell Out<br>5a. Failed or Unsuccessful Attempt to Reinsert Catheter<br>5b. Catheter related pain<br><br>PROBLEMS WITH SOLUTE/WATER CLEARANCE<br>6. Solute-Related<br>6a. Inadequate Clearance – Defined by Either Kt/V or Creatinine Clearance<br>6b. Inadequate Clearance – Phosphate Clearance<br>6c. Uraemic Symptoms/Poor Nutrition<br>6d. Clinical Signs of Poor Nutrition<br>6e. Hypoalbuminemia<br>6d. Loss of Residual Renal Function<br>6e. Patient Size<br>7. Fluid – UF-Related<br>7a. UF Failure – PET Defined<br>7b. Unable to Remove Excess Body Water<br>7c. Unwillingness to Prescribe More Dialysate Glucose to Achieve Sufficient UF<br>7d. Excess Fluid Removal	PERITONEAL LEAKS/HERNIA<br>8. Leaks<br>8a. Scrotal Oedema<br>8b. Pleuro-Peritoneal Leak<br>8c. Abdominal Wall<br>8d. Elsewhere<br>9. Hernia<br>9a. Inguinal<br>9b. Peri-Umbilical<br>9c. Elsewhere<br><br>PSYCHOSOCIAL/MEDICAL<br>10. Psychosocial<br>10a. Patient Choice/"Burnout"<br>10b. Depression<br>10c. Caregiver Choice/"Burnout"<br>10d. Change in Circumstance (e.g., Death of Caregiver, Change in Job, etc.)<br>11. Medical<br>11a. Physical Incapacity<br>11b. Mental Incapacity<br><br>RISK/DIAGNOSIS OF ENCAPSULATING PERITONEAL SCLEROSIS (EPS)<br>12a. Diagnosed EPS<br>13. Risk or Possibility of EPS<br>13a. Time on PD<br>13b. GI Symptoms but Not Formally Diagnosed with EPS<br><br>TRAINING RELATED (NOT FOR PATIENTS WHO HAVE BEEN ON PD)<br>15 Inability to retain training information<br>15a. Physical difficulty impairing ability to train (e.g. dexterity, visual impairment)<br>15b. Impairment of motivation to train to a sufficient standard<br><br>ADDITIONAL<br>16. Patient choice – change of modality selection	OTHER<br>14. Haemoperitoneum<br>14a. Intra-Abdominal Pathology<br>14b. Unexplained Cachexia/Failure to Thrive<br>999. Other Reason not Included Elsewhere

STROBE Statement—Checklist of items that should be included in reports of ***cohort studies***

	Item No	Recommendation	Page No
**Title and abstract**	1	(*a*) Indicate the study's design with a commonly used term in the title or the abstract	1,2,3
		(*b*) Provide in the abstract an informative and balanced summary of what was done and what was found	2
**Introduction**			
Background/rationale	2	Explain the scientific background and rationale for the investigation being reported	4
Objectives	3	State specific objectives, including any prespecified hypotheses	5
**Methods**			
Study design	4	Present key elements of study design early in the paper	5
Setting	5	Describe the setting, locations, and relevant dates, including periods of recruitment, exposure, follow-up, and data collection	5
Participants	6	(*a*) Give the eligibility criteria, and the sources and methods of selection of participants. Describe methods of follow-up	5
		(*b*) For matched studies, give matching criteria and number of exposed and unexposed	N/A
Variables	7	Clearly define all outcomes, exposures, predictors, potential confounders, and effect modifiers. Give diagnostic criteria, if applicable	6
Data sources/ measurement	8*	For each variable of interest, give sources of data and details of methods of assessment (measurement). Describe comparability of assessment methods if there is more than one group	Table S1
Bias	9	Describe any efforts to address potential sources of bias	6-7
Study size	10	Explain how the study size was arrived at	(in protocol)
Quantitative variables	11	Explain how quantitative variables were handled in the analyses. If applicable, describe which groupings were chosen and why	Table S1
Statistical methods	12	(*a*) Describe all statistical methods, including those used to control for confounding	6-7
		(*b*) Describe any methods used to examine subgroups and interactions	Supp Text
		(*c*) Explain how missing data were addressed	Supp Text
		(*d*) If applicable, explain how loss to follow-up was addressed	N/A

		(*e*) Describe any sensitivity analyses	N/A
**Results**			
Participants	13*	(a) Report numbers of individuals at each stage of study—eg numbers potentially eligible, examined for eligibility, confirmed eligible, included in the study, completing follow-up, and analysed	9
		(b) Give reasons for non-participation at each stage	Sup Text<br>SFig 1
		(c) Consider use of a flow diagram	SFig 1
Descriptive data	14*	(a) Give characteristics of study participants (eg demographic, clinical, social) and information on exposures and potential confounders	Table 1
		(b) Indicate number of participants with missing data for each variable of interest	Table 1
		(c) Summarise follow-up time (eg, average and total amount)	P9
Outcome data	15*	Report numbers of outcome events or summary measures over time	P9

Main results	16	(*a*) Give unadjusted estimates and, if applicable, confounder-adjusted estimates and their precision (eg, 95% confidence interval). Make clear which confounders were adjusted for and why they were included	P10<br>Sup T3
		(*b*) Report category boundaries when continuous variables were categorized	N/A
		(*c*) If relevant, consider translating estimates of relative risk into absolute risk for a meaningful time period	Fig S6
Other analyses	17	Report other analyses done—eg analyses of subgroups and interactions, and sensitivity analyses	P11
**Discussion**			
Key results	18	Summarise key results with reference to study objectives	P11
Limitations	19	Discuss limitations of the study, taking into account sources of potential bias or imprecision. Discuss both direction and magnitude of any potential bias	P12
Interpretation	20	Give a cautious overall interpretation of results considering objectives, limitations, multiplicity of analyses, results from similar studies, and other relevant evidence	P12
Generalisability	21	Discuss the generalisability (external validity) of the study results	P12, Sup Text
**Other information**			
Funding	22	Give the source of funding and the role of the funders for the present study and, if applicable, for the original study on which the present article is based	P14

*Give information separately for exposed and unexposed groups.

Note: An Explanation and Elaboration article discusses each checklist item and gives methodological background and published examples of transparent reporting. The STROBE checklist is best used in conjunction with this article (freely available on the Web sites of PLoS Medicine at http://www.plosmedicine.org/, Annals of Internal Medicine at http://www.annals.org/, and Epidemiology at http://www.epidem.com/). Information on the STROBE Initiative is available at http://www.strobe-statement.org.
